# NeuroStat: An Open-Source EEG Connectivity Platform for Randomised Controlled Trials

**DOI:** 10.3390/s26134019

**Published:** 2026-06-24

**Authors:** Usman Ghani, Iftikhar Ahmad, Shahbaz Pervez, Seyed Ebrahim Hosseini, Imran Khan Niazi

**Affiliations:** 1 Centre for Chiropractic Research, New Zealand College of Chiropractic, Auckland 1060, New Zealand; usman.ghani@nzchiro.co.nz (U.G.); imran.niazi@nzchiro.co.nz (I.K.N.); 2 Department of Information Technology, Faculty of Computing and Information Technology, King Abdulaziz University, Jeddah 21589, Saudi Arabia; 3 School of Applied IT, Whitecliffe, Auckland 1010, New Zealand; shahbazp@whitecliffe.ac.nz (S.P.); seyedh@whitecliffe.ac.nz (S.E.H.); 4 Health and Rehabilitation Research Institute, Auckland University of Technology, Auckland 1010, New Zealand; 5 Centre for Sensory-Motor Interaction, Department of Health Science and Technology, Aalborg University, 9220 Aalborg, Denmark

**Keywords:** electroencephalography, functional connectivity, phase lag index, randomised controlled trials, artefact removal, source localisation, open-source software, biomedical signal processing, clinical neurophysiology, Python

## Abstract

**Background:** Electroencephalographic (EEG) functional connectivity analysis requires multiple signal-processing, source-modelling, and statistical steps that can limit its adoption in clinician-led randomised controlled trials (RCTs). NeuroStat was developed as a prototype research tool to integrate this workflow; formal usability validation with clinician end-users has not yet been conducted. **Methods:** NeuroStat is an open-source Python/PyQt6 desktop application that integrates automated artefact removal (a Generalised Eigenvalue Decomposition for Artefact Identification [GEDAI] pathway and a traditional Artefact Subspace Reconstruction (ASR)/Independent Component Analysis (ICA)/ICLabel pathway), boundary element model (BEM) source localisation using the Desikan–Killiany atlas (68 cortical regions), Phase Lag Index (PLI) connectivity estimation across five canonical frequency bands, and RCT-oriented statistical analysis. Evaluation separated sensor-space and source-space claims: a sensor-level simulation (repeated across five independent random seeds) tested preprocessing robustness, a repeated source-space simulation tested recovery of a known cortical parcel-pair contrast after forward projection and inverse reconstruction, a PhysioNet benchmark tested posterior Desikan–Killiany alpha PLI in 20 healthy adults, and an illustrative application to 20 sessions from a published chiropractic RCT demonstrated real-world workflow applicability. **Results:** In the sensor-level simulation benchmark, the Traditional pathway achieved a mean absolute error of 0.168 ± 0.017 PLI units and root mean squared error of 0.219 ± 0.045 (mean ± SD across five independent random seeds) across all artefact conditions. In the source-space simulation, reconstructed alpha PLI for the known bilateral lateral-occipital parcel pair exceeded anterior control edges across 60 repeated condition runs (mean known-control difference = 0.105 PLI units, 95% CI 0.096–0.114; *t*(59) = 22.61, *p* < 0.001). In the PhysioNet source-space benchmark, posterior Desikan–Killiany alpha PLI was higher during eyes-closed than eyes-open rest (Cohen’s *d* = 0.85, *p* = 0.001; 16/20 subjects showing the expected direction) after ICLabel-enabled preprocessing. In the pilot RCT application, all 20 sessions completed processing without manual intervention, with default-mode network alpha PLI showing a pre-to-post change of +0.071 in the intervention group versus +0.015 in the active control group. **Conclusions:** NeuroStat integrates preprocessing, source-space construction, connectivity estimation, and statistical reporting within a parameter-logged desktop workflow for EEG functional connectivity studies. Current evidence supports initial technical feasibility, sensor-level preprocessing robustness for one pathway in controlled simulations, source-space recovery of a known parcel-level contrast, source-space sensitivity to an expected posterior alpha resting-state contrast, and error-free processing across 20 real RCT sessions in a pilot workflow demonstration. Formal usability testing, test–retest reliability analysis, participant-specific source-model validation, and clinical-population validation remain necessary before clinician-facing or trial-deployment claims can be made.

## 1. Introduction

Electroencephalography (EEG) offers distinct advantages for clinical trial outcome measurement: non-invasive recording, millisecond temporal resolution, portability, and substantially lower cost than functional MRI or PET [1]. These properties suit the repeated-measurement designs central to randomised controlled trials (RCTs), where session-to-session comparisons of neurophysiological state are required to evaluate intervention effects [2]. Despite the widespread availability of clinical EEG systems, neurophysiological outcome measures remain largely absent from trials conducted by allied health clinicians, physiotherapists, and chiropractors [3].

The barrier is not equipment access but analytical complexity. Extracting valid functional connectivity estimates from scalp EEG demands the following: bad channel detection and interpolation; spatial filtering to suppress artefacts; bandpass filtering with attention to phase distortion; re-referencing to minimise common-mode contamination; source reconstruction to address volume conduction; and phase-based connectivity estimation across anatomically defined regions [4,5,6]. Each step involves parameter decisions with downstream consequences, requiring proficiency in signal processing theory, programming (Python or MATLAB), and neuroanatomical parcellation. Acquiring these computational skills requires substantial time investment and usually depends on collaboration with biomedical engineers whose timelines and priorities may not align with clinician-led research workflows [3].

Contemporary open-source platforms, such as EEGLAB [7], MNE-Python [8], Brainstorm [9], and FieldTrip [10], provide comprehensive functionality for expert users but remain difficult for users without programming backgrounds. EEGLAB requires MATLAB and familiarity with scripting to extend beyond preset pipelines. MNE-Python operates primarily through command-line scripts. Brainstorm offers a more complete graphical environment but still demands substantive conceptual knowledge of forward modelling and inverse solutions. Automated preprocessing pipelines such as the Preprocessing Pipeline for EEG (PREP) [11], the Harvard Automated Processing Pipeline for Electroencephalography (HAPPE) [12], and similar tools address artefact removal but do not extend to source reconstruction or connectivity estimation. Critically, none of these tools integrates the complete workflow within an interface explicitly designed to reduce programming burden across the full pipeline.

Beyond signal processing barriers, clinician-led RCTs require managing fragmented infrastructure: separate systems for participant tracking, randomisation, EEG acquisition, signal analysis, statistical testing, and output generation [3,13]. This fragmentation introduces opportunities for transcription error, version control failures, and inconsistent preprocessing across sessions.

NeuroStat was developed as a prototype research tool with the goal of reducing scripting demands by integrating the EEG connectivity workflow within a graphical interface. The application links raw data import, preprocessing, source localisation, source-level PLI connectivity, statistical analysis, and publication-ready outputs. Development followed user-centred design principles informed by semi-structured consultations with practising clinicians, but formal usability testing with clinician end-users has not yet been completed. Two preprocessing pathways (GEDAI and traditional ASR/ICA/ICLabel) support different data characteristics and enable cross-method comparison. The application is freely available at https://github.com/ghani097/NeuroStat-for-RCTs (accessed on 21 June 2026).

Three formal evaluation experiments address distinct claims: (1) can automated preprocessing preserve known sensor-level connectivity patterns under controlled artefact contamination; (2) can the source-space construction workflow recover a known parcel-level coupling pattern after forward projection to scalp EEG and inverse reconstruction; and (3) does source-space analysis on real human EEG show the expected posterior alpha contrast between eyes-open and eyes-closed rest in healthy adults? In addition, an illustrative demonstration on RCT-structured data (a pre–post, two-group chiropractic trial subsample) shows that the complete workflow runs end-to-end on a realistic clinical-trial dataset; this demonstration is presented as a usability illustration rather than as a formal validation experiment.

## 2. Materials and Methods

### 2.1. Software Architecture and Design

**Figure 1:**
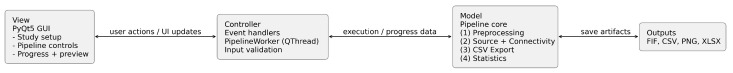
NeuroStat application architecture following the model-view-controller (MVC) design pattern. The View (PyQt6 GUI) handles study configuration, pipeline controls, and progress display. The Controller routes user actions to pipeline execution via a QThread worker. The Model executes sequential pipeline stages and writes output artefacts (FIF, CSV, PNG, and XLSX).

NeuroStat was implemented in Python 3.10, leveraging its mature scientific computing ecosystem and active neuroimaging community support [8]. The application follows a model-view-controller (MVC) architecture (Figure 1): the graphical interface is implemented in PyQt6 (View); event-handler functions manage user interaction and input validation (Controller); and the processing pipeline runs within a dedicated QThread (Model), maintaining interface responsiveness during computationally intensive EEG processing. Table 1 summarises the core technology stack.

**Figure A1:**
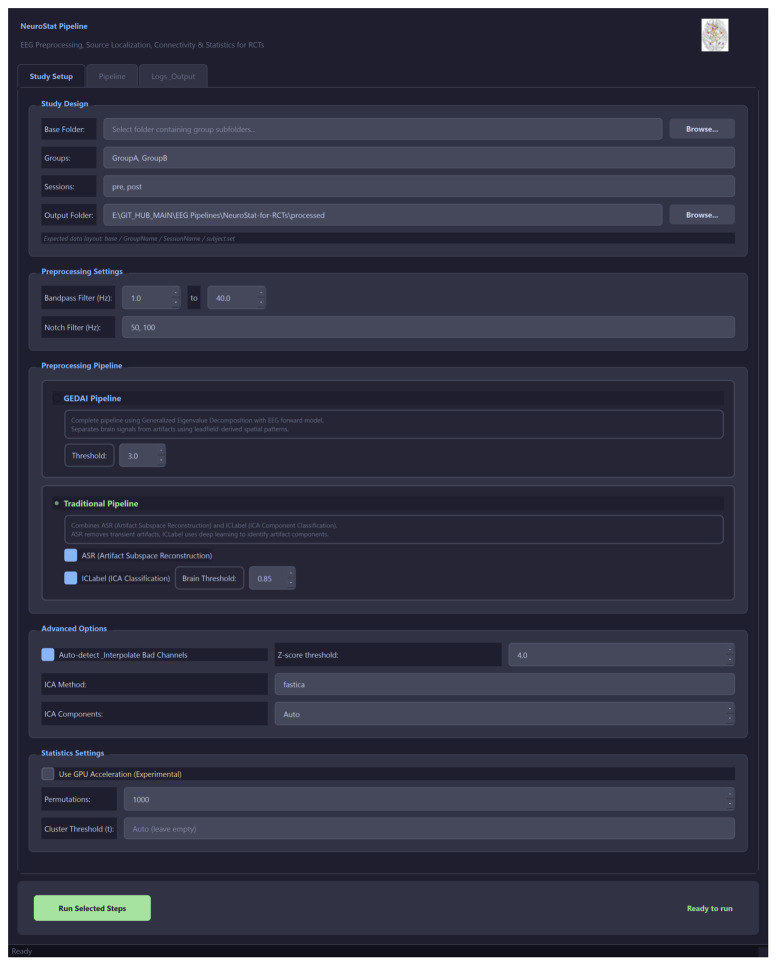
NeuroStat graphical user interface, Study Setup tab, showing study design fields (group names, session labels, output directory), preprocessing settings (bandpass/notch filters), pipeline selection (GEDAI or Traditional), and advanced options including ICA method and component count.

**Figure A2:**
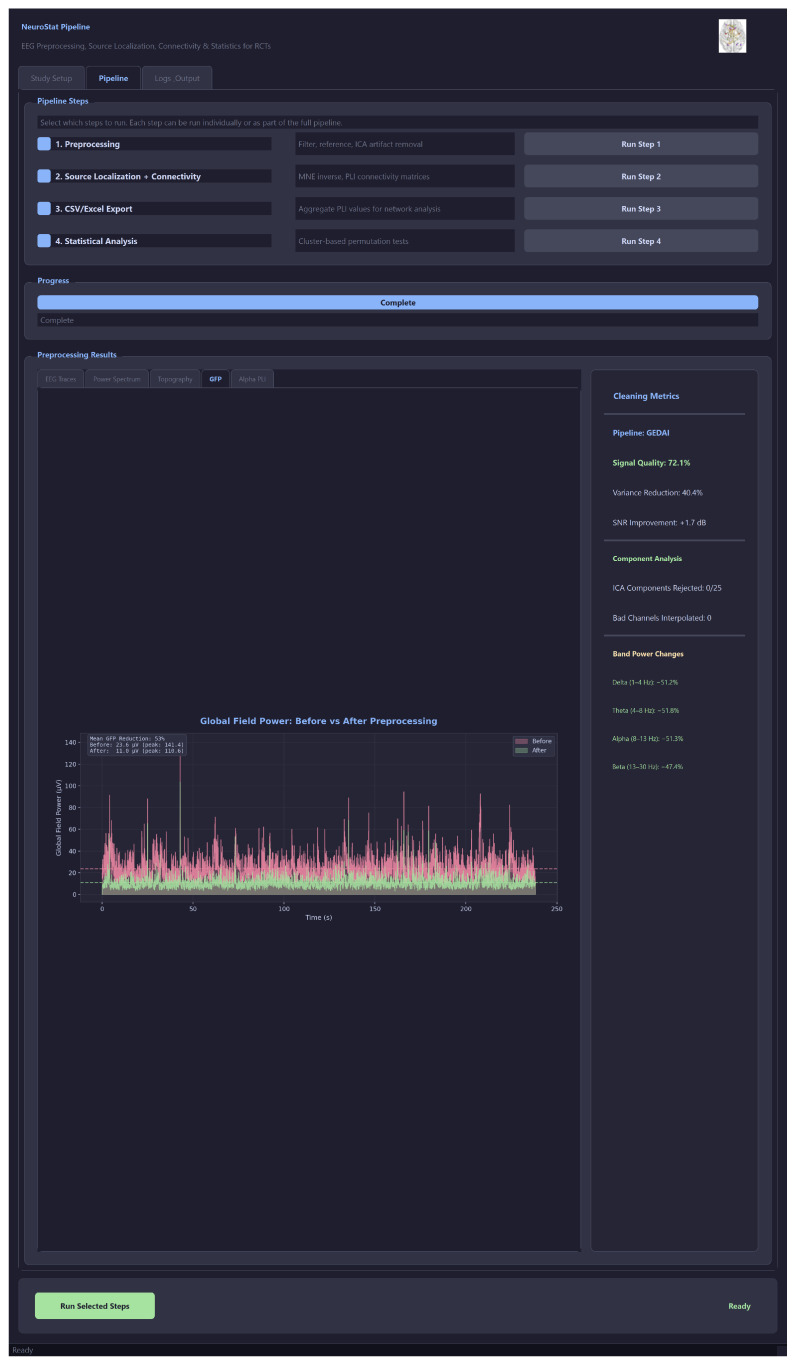
NeuroStat graphical user interface, Pipeline tab after a completed run, displaying the Global Field Power (GFP) plot (before and after preprocessing) and the Cleaning Metrics panel with signal quality score, variance reduction, SNR improvement, component rejection count, and per-band power changes.

**Figure A3:**
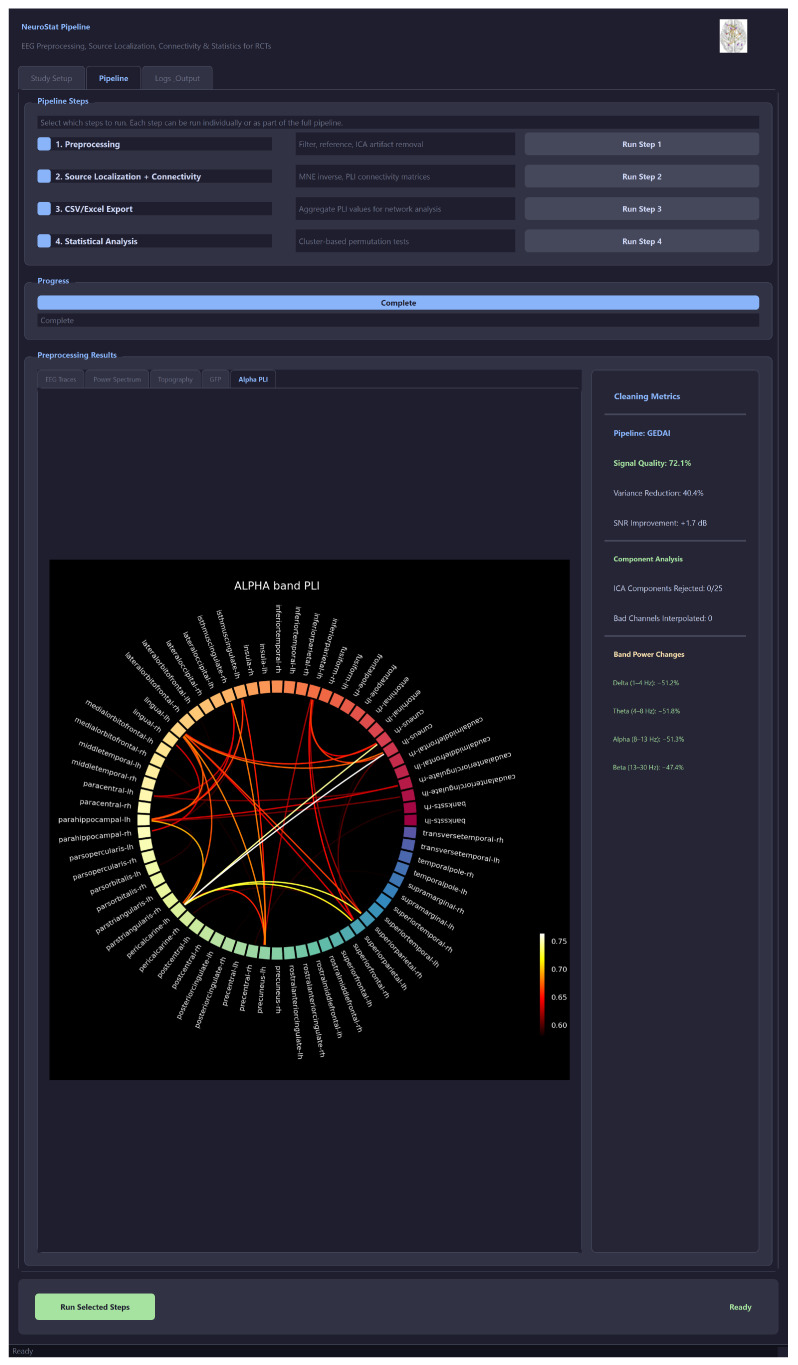
NeuroStat graphical user interface, Pipeline tab showing the alpha-band Phase Lag Index circular connectogram for a representative participant, with connection strength encoded by line weight and colour.

Development followed user-centred design principles [14,15]. Initial requirements were gathered through semi-structured interviews with practising chiropractors, neurologists, and clinical researchers. Five requirements were consistently identified: minimal technical prerequisites; automated preprocessing with sensible defaults; outputs interpretable without specialist neuroimaging training; support for pre–post and between-group RCT designs; and export formats suitable for submission. Development proceeded iteratively, with prototype versions evaluated by a clinician panel whose feedback shaped interface layout, contextual help features, and quality control reporting. These consultations informed design but were not a formal usability study; no task-completion, error-rate, or think-aloud data were collected. The study configuration module captures experimental parameters through a guided three-step wizard: (1) study design specification (group names, session labels, file format, and output directory); (2) preprocessing pathway and parameter selection (pipeline choice, filter settings, and ICLabel threshold); and (3) connectivity and statistics options (frequency bands and permutation count). All settings are stored in a JSON configuration file to enable exact reproduction of analyses. The manuscript evaluates a tagged development snapshot, release v1.0.0, corresponding to repository commit 4e1b6eb (https://github.com/ghani097/NeuroStat-for-RCTs/commit/4e1b6eb, accessed on 21 June 2026). Screenshots of the graphical interface are provided in Appendix A (Figure A1, Figure A2 and Figure A3).

### 2.2. Automated Preprocessing Pipeline

**Figure 2:**
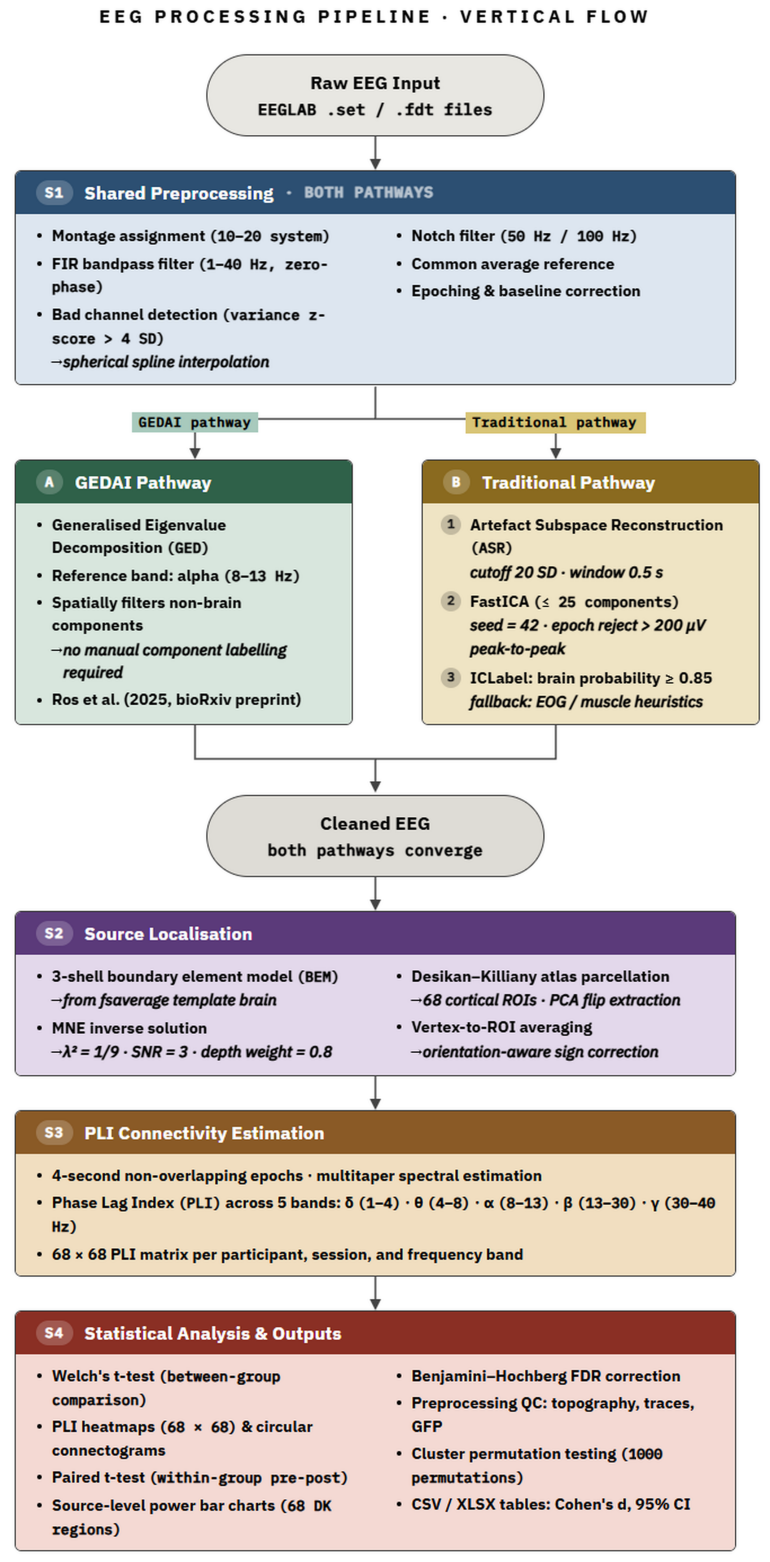
Default NeuroStat processing pipeline. Raw EEG files undergo shared preprocessing (Stage 1: montage assignment, bad channel detection and interpolation, bandpass and notch filtering, and average re-referencing) before diverging into one of two independent artefact-removal pathways. The GEDAI pathway applies a generalised eigenvalue decomposition using alpha-band activity as a brain-reference signal; the Traditional pathway applies ASR followed by FastICA and ICLabel component rejection. Both pathways produce cleaned EEG that enters the same source-localisation (Stage 2), PLI connectivity estimation (Stage 3), and statistical analysis and output generation (Stage 4) stages. Benchmark-specific short-recording modifications used in the PhysioNet analysis are described separately in Section 2.7.3. Individual default-pipeline stages are described in detail in Section 2.2, Section 2.3, Section 2.4, Section 2.5 and Section 2.6. Note: the text “Ros et al. (2025, bioRxiv preprint)” (reference [22]) appearing within the GEDAI pathway box is the algorithm reference, not a processing step; it is rendered in smaller italic type within the figure to distinguish it from the pipeline steps.

Figure 2 provides an overview of the default NeuroStat processing pipeline. NeuroStat implements two independent preprocessing pathways operating on the same raw EEG input, enabling method comparison and accommodating different data characteristics. Benchmark-specific short-recording adaptations are not shown in Figure 2 and are reported separately where used.

Traditional pathway. Raw EEG files in EEGLAB format (.set/.fdt) are loaded and electrode positions are assigned using the standard 10–20 montage. Bad channel detection applies variance-based z-score thresholding (threshold: 4 SD), with flagged channels reconstructed by spherical spline interpolation [16]. This step is conceptually similar to other automated statistical pipelines such as FASTER [17]. Spectral filtering uses a zero-phase FIR bandpass filter (1–40 Hz, firwin method in SciPy [18]) followed by notch filters at 50 Hz and 100 Hz. FIR filters preserve the phase relationships required for PLI computation [19]. Data are re-referenced to the common average. Artefact Subspace Reconstruction (ASR; cutoff 20 SD, window 0.5 s) [20] removes high-amplitude transients. The 20 SD cutoff corresponds to the conservative end of the range recommended for resting-state EEG [20], providing aggressive transient removal while minimising rejection of genuine neural activity; the 0.5 s window was selected to capture brief artefacts including eye blinks and muscle bursts. Independent Component Analysis (FastICA [21]) then decomposes the cleaned signal using up to 25 components (or *n* − 1 for *n* EEG channels, whichever was smaller). During ICA fitting, high-amplitude ICA-training segments exceeding 200 μV peak-to-peak amplitude were excluded to reduce the influence of extreme transients on the decomposition. This safeguard is applied before the later 4-s PLI epoching and should not be interpreted as the connectivity-epoch definition. The 200 μV threshold was selected after visual inspection of pilot high-amplitude transients during software development and has not been independently validated; users applying NeuroStat to populations with atypical EEG amplitude profiles (e.g., pathological or paediatric data) should treat this parameter as requiring data-specific calibration. FastICA was initialised with a fixed random state (seed = 42) to ensure deterministic component decomposition across repeated runs. Independent components were classified using ICLabel when available, and components with brain-class probability below 0.85 were rejected; if ICLabel was unavailable, ocular- and muscle-component heuristics were used as fallback criteria.

GEDAI pathway. Loading, bad channel detection, spectral filtering, notch filtering, and re-referencing are identical to the Traditional pathway. The GEDAI algorithm [22] then applies a generalised eigenvalue decomposition contrasting alpha-band (8–13 Hz) activity as a reference for brain-like spectral characteristics against broadband signal, spatially filtering components whose distributions deviate from cortically plausible patterns without requiring subjective component selection. At the time of writing, GEDAI remained available as a preprint; its formal peer-review status should therefore be confirmed before clinical deployment, and its implementation version should be verified against the peer-reviewed publication when available.

All preprocessing parameters are summarised in Table 2.

The 1–40 Hz passband removes slow drifts and high-frequency noise while retaining the physiological activity analysed here. Because the 40 Hz low-pass cutoff lies below both the 50 Hz mains frequency and its 100 Hz harmonic, the bandpass alone already attenuates line noise, so the 50 and 100 Hz notch filters are largely redundant in this configuration and are retained mainly as a safeguard for users who select a higher low-pass cutoff. Whether a requested notch is actually applied is governed by the Nyquist frequency (half the sampling rate) rather than by the low-pass cutoff: the pipeline discards any notch frequency at or above Nyquist and applies the remainder to the already band-limited signal. Consequently, with the default 1–40 Hz bandpass and the notch left enabled, recordings sampled at or above 200 Hz still have both the 50 and 100 Hz notches applied, but with no practical effect because the corresponding energy has already been removed by the low-pass. For the 160 Hz PhysioNet recordings (Nyquist 80 Hz), the 100 Hz notch is automatically omitted, and only the 50 Hz notch is applied, again with no practical effect on the retained 1–40 Hz signal.

### 2.3. Source Localisation

NeuroStat implements source localisation to estimate cortical activity from scalp-recorded potentials, addressing the volume conduction problem inherent in sensor-level connectivity estimation, namely the artificial inflation of coupling between nearby sensors caused by passive electrical spread through head tissues [23].

A three-shell boundary element model (BEM) is constructed from the fsaverage template brain using FreeSurfer [24]. Tissue conductivity values are 0.3, 0.006, and 0.3 S/m for scalp, skull, and brain compartments, respectively [25]. Source space comprises approximately 10,000 dipole sources per hemisphere at ico5 spacing in the default application pipeline. Here ico3 and ico5 indicate how densely candidate source points are placed on the cortical surface: ico3 uses about 1300 source points and ico5 about 20,000. The finer ico5 spacing gives greater spatial detail at higher computational cost, while the coarser ico3 is faster and is used when many recordings must be processed. Source-space construction proceeds in seven explicit steps: cleaned scalp EEG is assigned a standard electrode montage; the fsaverage BEM defines the head-volume conductor; a forward solution maps cortical dipoles to scalp sensors; an MNE inverse operator is estimated from the recording covariance (computed as the empirical sample covariance matrix across all available clean samples of the preprocessed EEG); source time series are reconstructed from the cleaned EEG; source estimates are parcellated into 68 Desikan–Killiany cortical regions; and representative parcel time courses are used to compute parcel-level PLI matrices. For each parcel, the principal component explaining maximum variance across the dipole source time courses within that parcel is extracted as the representative regional time course; sign ambiguity is resolved by aligning the component polarity to the majority dipole orientation within the parcel (PCA flip). Source activity is estimated using the Minimum Norm Estimation (MNE) inverse solution [26] with Tikhonov regularisation ($\lambda^{2}=1/9$; equivalent to the commonly used SNR = 3 setting) and depth weighting (exponent = 0.8), consistent with standard MNE-Python practice. Source activity is parcellated into 68 cortical regions using the Desikan–Killiany atlas [27], and representative region time courses are extracted via vertex-to-ROI averaging. This procedure constructs a source-space representation for connectivity analysis; it should not be interpreted as proof of exact anatomical localisation for an individual participant. Because source-space connectivity estimates remain sensitive to inverse-model choices and residual leakage, source localisation was interpreted as an enabling processing stage rather than as a validated ground truth transformation [28]. Template-based forward models can introduce centimetre-scale localisation errors relative to individual anatomy [29]; at the mean Desikan–Killiany parcel scale (approximately 2–3 cm diameter), this implies that individual parcels may contain contributions from adjacent cortical regions.

### 2.4. Functional Connectivity Estimation

PLI connectivity is computed between all 68 × 68 region pairs within five canonical frequency bands: delta (1–4 Hz), theta (4–8 Hz), alpha (8–13 Hz), beta (13–30 Hz), and low gamma (30–40 Hz) [2]. Frequencies below 1 Hz and above 40 Hz were not analysed because they fall outside the preprocessing passband. The PLI quantifies asymmetry in the phase-difference distribution between two signals:

(1) PLI=signImCxy(f)

where $C_{xy}\left(f\right)$ is the cross-spectral density at frequency *f*, Im denotes the imaginary part, and 〈·〉 denotes the time average [30]. In the multitaper implementation used here, Im(Cxy(f)) is the cross-spectral analogue of $\sin(\Delta \phi_{xy})$ from the analytic-signal formulation of Stam et al. [30]. PLI ranges from 0 (no consistent phase relationship) to 1 (perfect phase-locking with non-zero lag). Its insensitivity to zero-lag connectivity makes it appropriate for scalp EEG where volume conduction produces instantaneous coupling artefacts [31].

Region time courses are segmented into 4-s non-overlapping epochs with multitaper spectral estimation, yielding 68 × 68 PLI matrices per participant, session, and frequency band. This duration was selected to balance low-frequency spectral resolution against estimate stability, consistent with evidence that EEG connectivity metrics vary with epoch length [32]. At the lowest analysed frequency (1 Hz), a 4-s epoch provides a minimum of four complete cycles, which is generally considered the lower bound for reliable phase estimation.

### 2.5. Statistical Analysis

Between-group comparisons use Welch’s independent-samples *t*-test, which maintains correct Type I error control under heterogeneous variances. Within-group pre–post comparisons use paired *t*-tests. Both test selections assume approximate normality; prior to analysis, Shapiro–Wilk testing is applied to group-level PLI distributions, and a non-parametric equivalent (Mann–Whitney *U* for between-group; Wilcoxon signed-rank for pre–post) is substituted automatically if the normality assumption is violated (*p* < 0.05). Multiple comparison correction applies the Benjamini–Hochberg False Discovery Rate procedure [33] and cluster-based permutation testing (1000 permutations) [34]. Results are exported as formatted CSV and Excel tables with effect sizes (Cohen’s *d*) and 95% confidence intervals.

### 2.6. Visual Outputs

**Figure 3:**
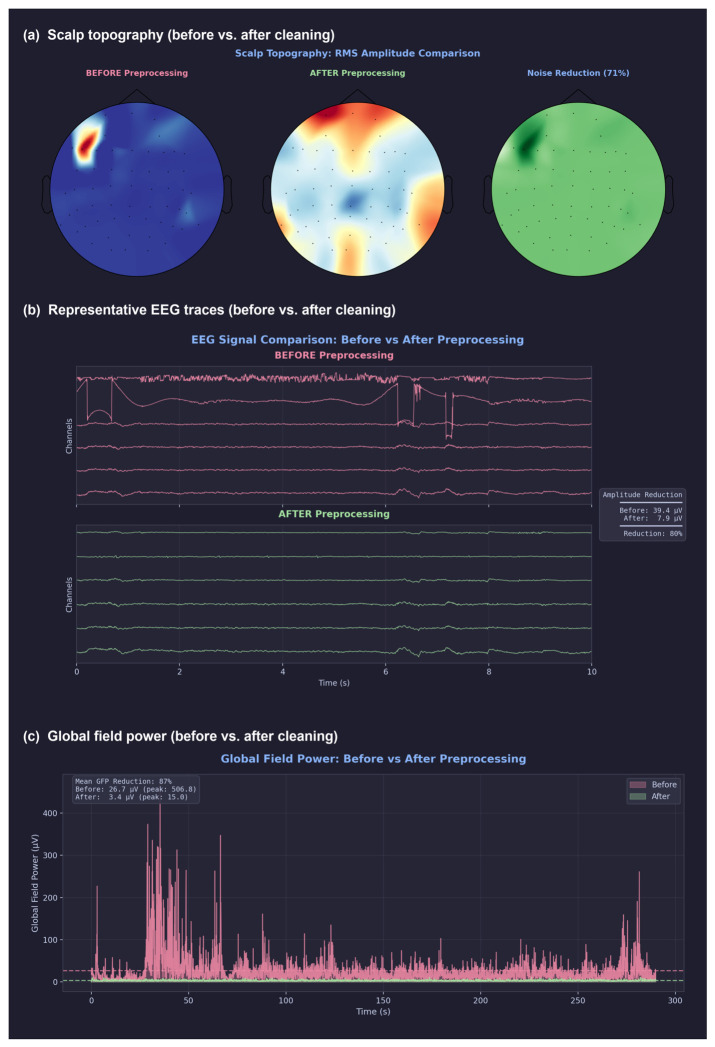
Preprocessing quality-control outputs generated from a representative EEG recording processed through the NeuroStat pipeline, shown as three stacked panels for legibility. (**a**) Scalp topography of RMS amplitude before and after cleaning, with the corresponding noise-reduction map. (**b**) Representative multi-channel EEG traces before and after cleaning. (**c**) Global field power across the recording before and after cleaning. Each panel is reproduced at full pipeline-output resolution.

**Figure 4:**
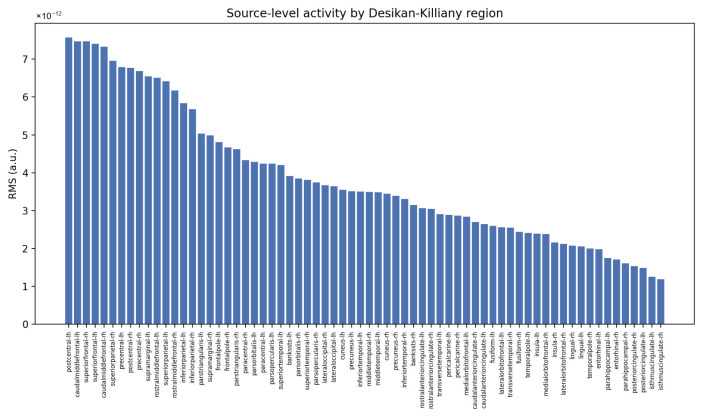
Source-level activity bar chart across 68 Desikan–Killiany cortical regions generated from a representative participant recording.

**Figure 5:**
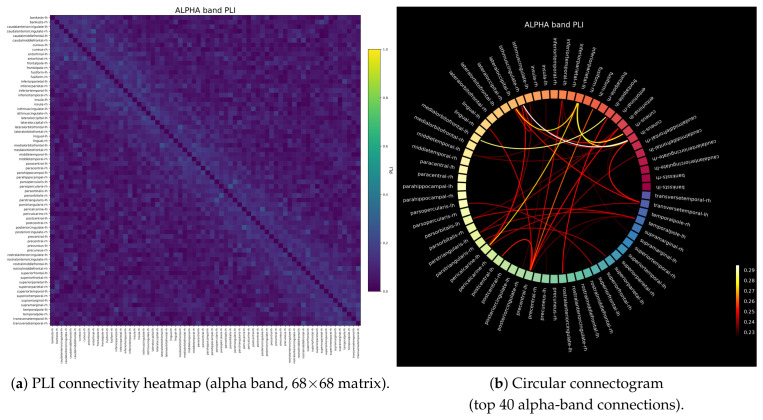
NeuroStat connectivity visualisations generated from a representative participant recording. (**a**) Alpha-band PLI connectivity heatmap (68 × 68). (**b**) Circular connectivity plot displaying the top 40 connections; line thickness scales with PLI magnitude.

NeuroStat generates four categories of visualisation (Figure 3, Figure 4 and Figure 5): (a) preprocessing quality plots displaying scalp topography, representative EEG traces, and global field power before and after cleaning; (b) source-level power bar charts summarising regional activity across the Desikan–Killiany atlas; (c) colour-coded 68 × 68 PLI heatmaps enabling identification of strongly connected region pairs; and (d) circular connectograms displaying the top connections with line thickness proportional to PLI magnitude. The previously used power spectral density panel was removed because it could be over-interpreted as a stand-alone validation metric. All outputs are exported in PNG, SVG, or PDF format and generated without post-processing intervention.

### 2.7. Validation Design

#### 2.7.1. Internal Validation: Simulated EEG

To evaluate preprocessing robustness under controlled conditions, 64-channel EEG was simulated at 250 Hz for 120 s using a BioSemi-64 montage. Background activity was modelled as spectrally shaped (1/f) pink noise. Three cross-regional coupling patterns were embedded using Kuramoto-style phase-coupled oscillators (Table 3). This benchmark was designed to test whether automated preprocessing preserved known sensor-level PLI structure under increasing artefact burden. It did not validate source localisation accuracy, atlas parcellation fidelity, leakage correction, or source-space connectivity recovery. GEDAI results are reported because GEDAI is an available NeuroStat pathway, but the simulated coupling patterns were not designed to test GEDAI’s leadfield-based design assumptions; GEDAI performance in this benchmark should therefore not be generalised beyond these known sensor-level patterns. Four artefact severity conditions were generated: clean (SNR = 10 dB, no artefacts), light (SNR = 5 dB; 10 blinks/min; 5 EMG bursts/min; 2 μV line noise), moderate (SNR = 5 dB; 30 blinks/min; 15 EMG bursts/min; 5 μV line noise), and heavy (SNR = 2 dB; 50 blinks/min; 30 EMG bursts/min; 10 μV line noise). Blink transients were modelled as Gaussian-shaped voltage steps with amplitudes spanning approximately 80–120 μV and duration of approximately 300 ms, applied to frontal channels with spatial fall-off; EMG bursts as bandpass-filtered (20–100 Hz) signals on temporal channels; and line noise as 50 Hz sinusoids with per-channel random phase. A deterministic random seed (42; chosen as a fixed arbitrary seed for exact reruns) ensured full reproducibility [35]. To assess simulation variability, the benchmark was repeated with five independent random seeds [42, 123, 456, 789, 1024] for both preprocessing pathways; the aggregate RMSE and MAE represent mean ± SD across all five seeds. Condition-wise results described in Section 3.2 use seed 42 as the primary reference. Each dataset was processed through both preprocessing pathways and recovery accuracy was quantified using RMSE and MAE between expected and recovered sensor-level PLI values across all 12 condition–pattern combinations. Pooled Pearson correlations were treated as descriptive only and not interpreted as primary metrics because each artefact-level panel comprised only three known patterns, yielding insufficient degrees of freedom for reliable correlation estimation. Additionally, the same three patterns were repeated across artefact levels, inducing statistical dependence that violates the independence assumption of standard correlation tests. Accordingly, RMSE and MAE were used as the primary recovery metrics.

#### 2.7.2. Source-Space Simulation Benchmark

To directly test source-space construction under known ground truth, a separate source-level simulation was added. Alpha activity with a fixed non-zero phase lag (10 Hz; phase lag π/3) was generated in bilateral lateral-occipital Desikan–Killiany parcels on the fsaverage cortical surface. Each simulated recording lasted 60 s at 160 Hz and was analysed as 15 non-overlapping 4 s epochs. The source signal was embedded at amplitude 2.0 arbitrary units against 1/f background source noise scaled to 0.005 arbitrary units before forward projection (approximate source-domain signal-to-background ratio 52 dB), with small Gaussian amplitude noise and slow phase jitter added to avoid a perfectly deterministic waveform. The simulated source activity was forward-projected to 62 scalp EEG channels using the same fsaverage BEM framework and then reconstructed with the NeuroStat BEM/MNE inverse pipeline using ico3 source spacing for computational tractability.

Three conditions were evaluated: clean, light artefact, and moderate artefact. Each condition was repeated 20 times with independent random seeds (60 reconstructed source-space runs total). The endpoint was reconstructed alpha PLI between the known lateral-occipital parcels, compared with eight anterior/non-posterior control edges linking the known parcels to rostral middle frontal and precentral control parcels. These controls were selected to provide anatomically distinct non-posterior comparator edges and were not distance matched. Condition-level uncertainty was summarised using 95% confidence intervals across repeats, and a one-sample *t*-test assessed whether the known-control PLI difference was greater than zero across all 60 source-space runs. This benchmark tests whether the source-space construction and parcellation workflow can recover a known parcel-level contrast under controlled conditions; it does not estimate localisation error relative to individual MRI anatomy.

#### 2.7.3. Source-Space Physiological Benchmark: PhysioNet EEGBCI Dataset and Procedure

**Figure 6:**
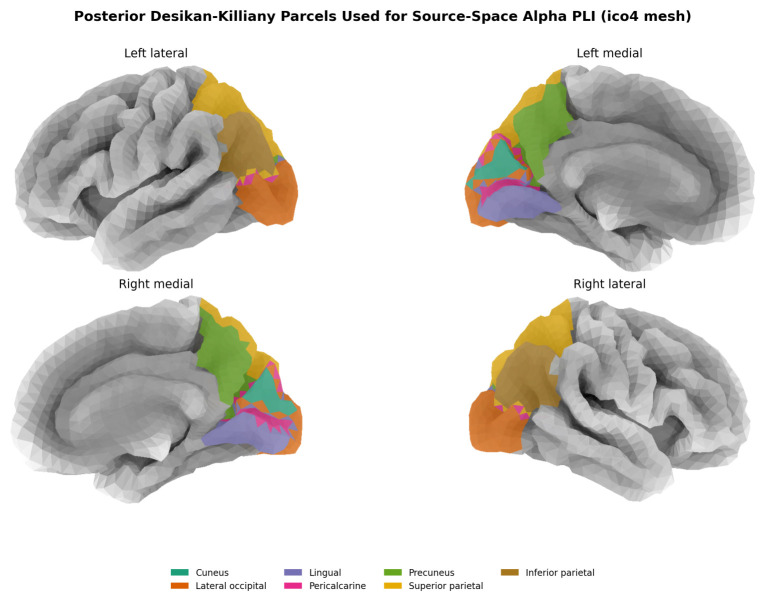
Posterior Desikan–Killiany source parcels used to define the PhysioNet source-space posterior alpha PLI endpoint. The Nilearn rendering shows the fsaverage aparc regions included bilaterally: cuneus, lateral occipital, lingual, pericalcarine, precuneus, superior parietal, and inferior parietal parcels. Colours identify individual posterior parcels on the downsampled fsaverage surface mesh used for display.

To confirm sensitivity to genuine physiological connectivity differences in source space, NeuroStat was applied to 20 subjects (S001–S020) from the publicly available PhysioNet EEG Motor Movement/Imagery Database [36,37], which provides 64-channel EEG recorded at 160 Hz. Eyes-open (Run 01) and eyes-closed (Run 02) one-minute baseline recordings were processed using the Traditional pathway with parameters adapted for short recording duration. The primary endpoint was mean source-space posterior alpha PLI averaged across bilateral posterior Desikan–Killiany parcels (Figure 6): cuneus, lateral occipital, lingual, pericalcarine, precuneus, superior parietal, and inferior parietal regions [27,38]. Source time courses were estimated with the BEM/MNE pipeline described above and parcellated before PLI estimation. Other frequency bands and anatomical regions were not tested. Normality of paired differences was checked with Shapiro–Wilk testing. A two-tailed paired *t*-test was specified as the primary test, with Wilcoxon signed-rank testing retained as a sensitivity analysis. Effect size was quantified as paired-samples Cohen’s *d*.

##### Pipeline Modifications for Short Recordings

The PhysioNet benchmark used a short-recording-adapted configuration rather than the default NeuroStat configuration. The ICA decomposition was limited to 15 components because each run contained only one minute of data. ICLabel was active, and the ICLabel brain-probability threshold was relaxed from the default 0.85 to 0.50 to reduce over-rejection in short recordings. ASR was disabled because these one-minute recordings did not provide an extended clean calibration segment. Source-space connectivity was computed from 2.0-s epochs with 50% overlap to increase the number of analysable windows. The source-space benchmark used ico3 source spacing for computational tractability across all 40 short PhysioNet recordings, whereas the default application pipeline uses ico5 spacing. Because epoch length and source-space discretisation affect EEG connectivity estimates [32], PLI values obtained from this PhysioNet benchmark should not be directly compared numerically with 4.0-s simulation benchmark values or default-pipeline outputs. Accordingly, this benchmark evaluates a short-recording source-space configuration and is interpreted within-benchmark only. Each modification was driven by the 60-s recording constraint: reduced ICA dimensionality (15 components) prevents overfitting on limited data; ASR was disabled because the short duration does not provide an adequate clean-segment calibration window; the relaxed ICLabel threshold (0.50) avoids over-rejection when few components are available; and 2.0-s overlapping epochs are necessary to obtain sufficient spectral estimates. These adaptations collectively represent a configuration appropriate for brief recordings and should not be interpreted as indicative of default NeuroStat preprocessing performance on typical clinical or research recordings.

The rationale for this benchmark is that increased posterior alpha connectivity during eyes-closed versus eyes-open rest is one of the most robustly replicated findings in EEG research [38,39], providing a clear directional criterion against which automated source-space pipeline sensitivity can be evaluated.

#### 2.7.4. Method Comparison on Real EEG

Both preprocessing pathways were applied to the same 20 PhysioNet subjects using the same eyes-closed versus eyes-open endpoint in the existing posterior-channel comparison script. These sensor-level method-comparison results are reported only as a preprocessing sensitivity check and are not used as source-space validation evidence.

#### 2.7.5. Illustrative Application: Real RCT-Structured Data Acquisition and Processing

To illustrate workflow applicability to genuinely RCT-structured data, NeuroStat was applied to a subset drawn from a published chiropractic randomised controlled trial [40]. Five participants from each group (GroupY: chiropractic HVLA manipulation; GroupZ: active control) were selected as a convenience subsample for a workflow demonstration only; this subsample was not powered for statistical inference. Resting-state EEG (64 channels, 2048 Hz) was recorded at two time points: a pre-intervention baseline and a post-intervention follow-up. The full trial enrolled *n* = 76 adults with chronic low-back pain and documented neuroplastic responses across pain, mood, sleep, and quality of life outcomes [40]; the present subset was drawn solely to verify that NeuroStat processes data with a genuine two-group, two-timepoint structure without error, and to demonstrate that the direction of any observed alpha-band PLI change is consistent with the neuroplastic trends reported in the source trial. Resting-state data were used exclusively; somatosensory-evoked potential recordings from the same trial were not included.

Raw FieldTrip MATLAB v7.3 HDF5 .mat files were converted to EEGLAB .set format using a provided helper script (convert_ft_to_eeglab.py; available in the repository) that reads trial arrays and channel labels via h5py and exports via MNE’s EEGLAB exporter. All 20 recording sessions (5 subjects × 2 groups × 2 timepoints) were processed through the NeuroStat GEDAI pathway with default settings: no parameter modifications relative to the default GEDAI configuration. The primary reporting endpoint was mean source-space alpha-band PLI for posterior Desikan–Killiany parcels (identical ROI definition to the PhysioNet benchmark), and the pre-to-post direction of change was compared qualitatively with the full-trial published result. This subsection constitutes a workflow demonstration, not a replication of the original trial findings; statistical comparisons between groups or timepoints in this subsample are not reported. Table 4 summarises the specific validation claims evaluated in this manuscript and the limitations that remain for each.

## 3. Results

### 3.1. Quality Verification of Simulated Data

Diagnostic inspection confirmed that simulated datasets exhibited the intended properties. Spectral inspection revealed clearly identifiable peaks at 6, 10, and 20 Hz in the clean condition, corresponding to the three known oscillatory frequencies, and progressively broader spectra under heavier artefact conditions. The sensor-level alpha-band PLI matrix for the clean dataset showed a pronounced frontal–parietal block structure corresponding to the known strong alpha coupling (expected PLI = 0.80), with cross-regional sub-matrix values ranging from 0.65 to 0.82. These diagnostics confirmed that the simulation framework produced data with a known sensor-space connectivity structure suitable for benchmarking preprocessing robustness [35].

Source localisation: computational feasibility only. Source localisation executed successfully for all eight simulated condition–method runs, producing 68-region source time courses and five source-level PLI matrices per run. These outputs demonstrate that the source-localisation workflow completed on the simulated data, but they were not used as accuracy metrics because no source-space ground truth was available. The source-level outputs therefore represent computational feasibility, not validation of anatomical localisation or source-space connectivity.

### 3.2. Recovery of Known Connectivity

The simulation benchmark tested preprocessing robustness at the sensor level. Source-level connectivity recovery was not evaluated because the known patterns were defined on simulated sensor channels rather than anatomical cortical sources. Table 5 summarises sensor-level connectivity recovery across all 12 condition–pattern combinations. The Traditional pathway achieved RMSE =0.219±0.045 and MAE =0.168±0.017 PLI units (mean ± SD across five seeds), whereas the GEDAI pathway yielded RMSE =0.422±0.012 and MAE =0.373±0.010 (mean ± SD across the same five seeds). The negligible GEDAI SD confirms that its uniform underestimation is seed-independent and not an artefact of a single random initialisation. The Traditional pathway achieved smaller absolute error in all 12 condition–pattern combinations, but GEDAI’s weaker performance should be interpreted within the limits of a benchmark that was not designed around GEDAI’s leadfield-based assumptions.

The GEDAI result in Table 5 should not be generalised beyond these known sensor-level patterns because the simulation was not designed to test GEDAI’s leadfield-based assumptions; GEDAI’s weaker performance in this benchmark reflects an incompatibility between the known coupling topology and GEDAI’s design, not a general deficiency of the algorithm.

Condition-wise analysis showed that Traditional pathway error remained limited across all artefact levels: MAE = 0.095 (clean), 0.250 (light), 0.120 (moderate), and 0.143 (heavy). The weak theta pattern (expected PLI = 0.30) was recovered within 0.04–0.09 PLI units, demonstrating sensitivity to modest coupling magnitudes. The strong alpha pattern (expected PLI = 0.80) was well recovered under clean conditions (recovered = 0.744; error = 0.056) but attenuated under heavy contamination (recovered = 0.586; error = 0.214). These observations support the Traditional pathway as the best-supported option in the current benchmark while also showing that error increased with contamination rather than remaining invariant.

GEDAI consistently underestimated known PLI values (recovered 0.12–0.19 across all conditions regardless of expected value). Because the simulated coupling patterns used sinusoidal oscillators with spatial topographies that were not constrained by realistic lead-field projections, the benchmark was not designed to test GEDAI’s design assumptions. The weaker GEDAI performance in this benchmark should therefore not be generalised beyond these specific known patterns. Users should select preprocessing pathways based on data characteristics and cross-method comparison on their own datasets.

**Figure 7:**
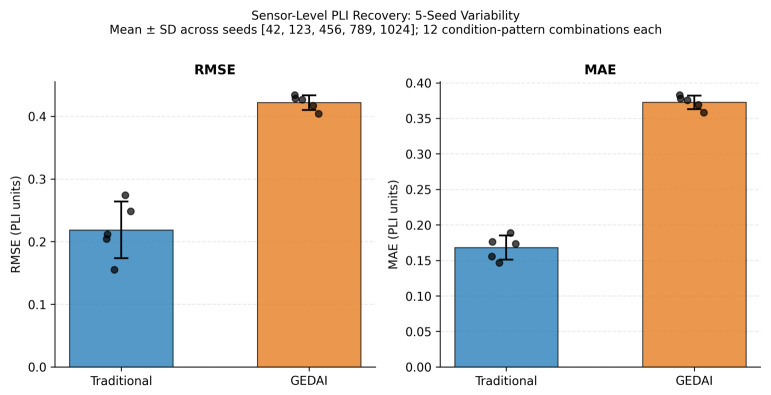
Sensor-level connectivity recovery error across five independent random seeds [42, 123, 456, 789, 1024] for the Traditional and GEDAI preprocessing pathways. Bars show mean RMSE (**left**) and MAE (**right**); error bars show standard deviation across seeds. The Traditional pathway shows moderate seed-to-seed variability (RMSE SD =0.045), whereas the GEDAI pathway’s negligible SD (RMSE SD =0.012) confirms that its uniform underestimation is seed-independent and not an artefact of a single random initialisation.

**Figure 8:**
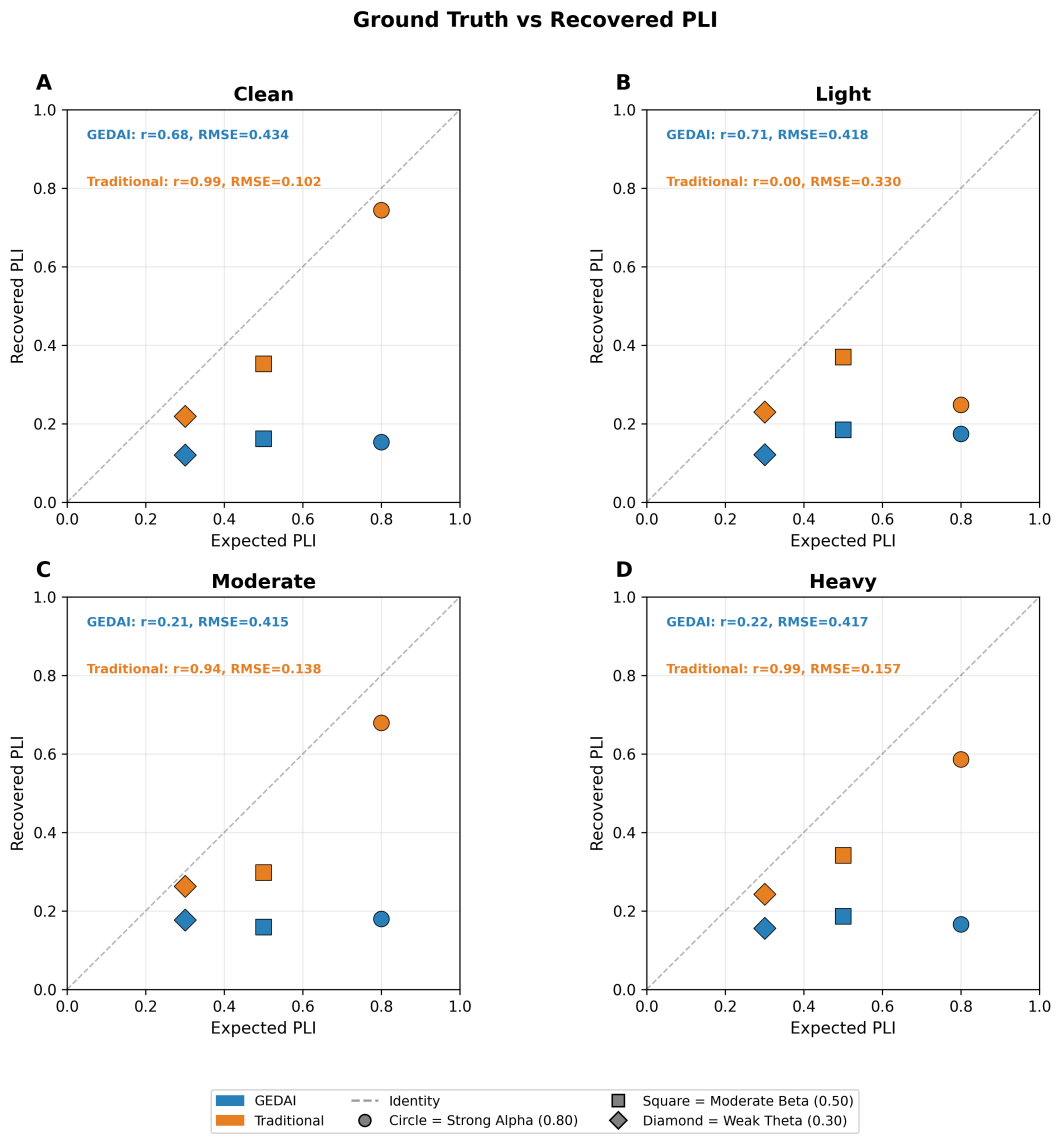
Ground truth (expected) versus recovered sensor-level PLI for the GEDAI (blue) and Traditional (orange) preprocessing pathways across four artefact severity conditions: (**A**) clean, (**B**) light artefact, (**C**) moderate artefact, and (**D**) heavy artefact. Marker shapes indicate the connectivity pattern: circles (∘) = strong alpha (10 Hz, expected PLI =0.80); squares (□) = moderate beta (20 Hz, expected PLI =0.50); diamonds (⋄) = weak theta (6 Hz, expected PLI =0.30). The dashed diagonal denotes perfect recovery. The Traditional pathway showed closer correspondence with known values than the GEDAI pathway across all conditions. GEDAI consistently underestimated all patterns to 0.12–0.19 regardless of expected PLI, reflecting an incompatibility between the sinusoidal coupling topology and GEDAI’s leadfield-based assumptions (see text). This benchmark evaluates sensor-level preprocessing robustness only; source localisation, atlas parcellation, leakage correction, and source-space connectivity recovery were not validated.

**Figure 9:**
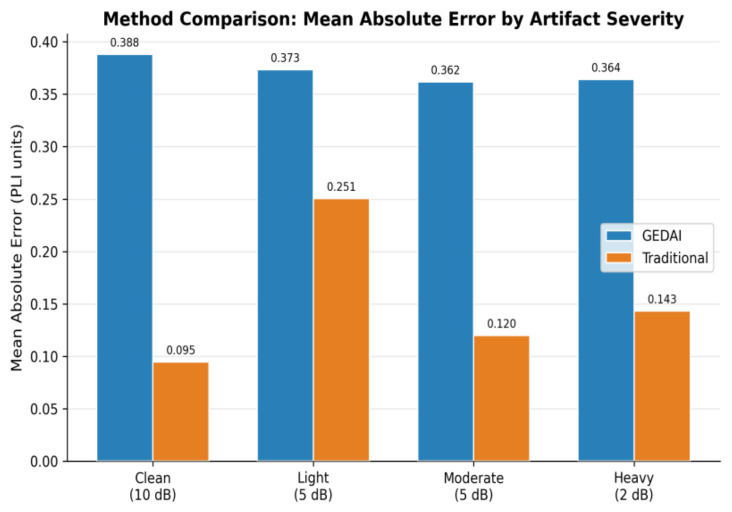
Mean absolute error (PLI units) by artefact severity condition for GEDAI and Traditional preprocessing pathways. Lower values indicate more accurate recovery of known sensor-level connectivity patterns. The Traditional pathway outperformed the GEDAI pathway at all artefact levels. This benchmark evaluates sensor-level preprocessing robustness only; source localisation, atlas parcellation, leakage correction, and source-space connectivity recovery were not validated.

Figure 7 shows RMSE and MAE with SD error bars across the five seeds. Figure 8 shows ground truth versus recovered PLI values across the four artefact conditions. Figure 9 illustrates MAE by artefact severity for both pathways.

### 3.3. Source-Space Simulation Recovery

**Figure 10:**
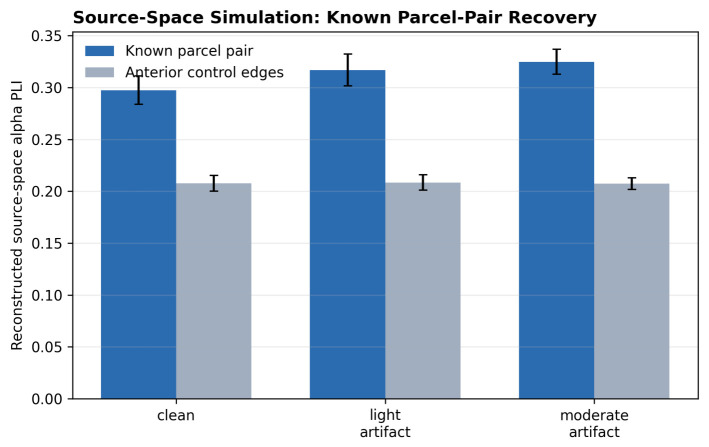
Source-space simulation recovery. Alpha-band phase-lagged activity was generated in bilateral lateral-occipital Desikan–Killiany parcels, forward-projected to scalp EEG, reconstructed using the BEM/MNE source-space pipeline, parcellated, and analysed with parcel-level PLI. Bars show mean reconstructed PLI for the known parcel pair and anterior control edges across 20 repeats per condition; error bars show 95% confidence intervals.

The source-space simulation tested the full source construction chain: cortical source generation in known Desikan–Killiany parcels, BEM forward projection to scalp EEG, MNE inverse reconstruction, parcellation, and parcel-level PLI estimation. Across 60 repeated source-space runs, reconstructed alpha PLI for the known bilateral lateral-occipital parcel pair exceeded anterior control edges (Figure 10; mean known-control difference = 0.105 PLI units, 95% CI 0.096–0.114; *t*(59) = 22.61, *p* < 0.001, Cohen’s *d* = 2.92). Condition-level known-control differences were 0.090 (95% CI 0.074–0.105) in the clean condition, 0.108 (95% CI 0.091–0.126) in the light-artefact condition, and 0.117 (95% CI 0.102–0.133) in the moderate-artefact condition. These results support recovery of a known source-space parcel-level contrast after forward projection and inverse reconstruction, while not eliminating the general limitations of template-based source localisation. The known edge was not consistently among the top 5% of all reconstructed parcel-pair edges, so this benchmark supports contrast recovery against prespecified controls rather than whole-connectome edge ranking.

### 3.4. Source-Space Physiological Benchmark: PhysioNet EEGBCI

Using a short-recording-adapted source-space pipeline configuration with ICLabel active, mean posterior Desikan–Killiany alpha PLI was significantly higher during eyes-closed rest (*M* = 0.133, *SD* = 0.019) than eyes-open rest (*M* = 0.114, *SD* = 0.013): *t*(19) = 3.79, *p* = 0.001, Cohen’s *d* = 0.85. Shapiro–Wilk testing did not indicate non-normality of paired differences (*W* = 0.970, *p* = 0.761), so the paired *t*-test was retained as the primary test; the Wilcoxon sensitivity analysis gave the same inference (*W* = 24.0, *p* = 0.001). Sixteen of twenty participants (80%) showed the expected eyes-closed > eyes-open direction; the four reversed subjects were S002, S006, S008, and S019. These source-space posterior alpha PLI values were computed from 2.0-s overlapping epochs and should not be compared numerically with 4.0-s simulation benchmark values.

ICLabel was applied successfully in all eyes-open and eyes-closed recordings. Across the 300 classified eyes-open components, ICLabel assigned 82 as brain, 36 as eye blink, 34 as muscle artefact, 6 as heart beat, 4 as channel noise, and 138 as other. Across the 300 eyes-closed components, 162 were classified as brain, 22 as eye blink, 17 as muscle artefact, 2 as channel noise, and 97 as other. ICA component rejection was numerically higher during eyes-open recordings (EO: 2.25 ± 1.59 components; EC: 1.45 ± 1.19 components), but the paired comparison did not reach the conventional 0.05 threshold (*t*(19) = 1.93, *p* = 0.068); this should be interpreted as inconclusive rather than evidence of equivalence. Frontal residual variance after preprocessing was higher in eyes-open than eyes-closed recordings (1503.6 vs. 464.7 μV^2^; paired *p* = 0.020), and the frontal transient proxy was also higher in eyes-open recordings (15.65 vs. 6.95 events; paired *p* = 0.004). A sensitivity analysis excluding subjects with large EO–EC ICA rejection differences (≥3 components; S002, S004, S012) retained the source-space effect (*n* = 17, *t*(16) = 3.61, *p* = 0.002, Cohen’s *d* = 0.88; 14/17 in the expected direction). Table 6 summarises the source-space benchmark statistics. These results are directionally consistent with the prior eyes-closed versus eyes-open EEG literature [38,39], but no direct manual-analysis comparator on the same 20 subjects was available. It should be noted that eyes-open and eyes-closed recordings were obtained from fixed runs (Run 01 and Run 02, respectively), meaning that condition and recording order were not fully counterbalanced. The higher frontal residual variance observed in eyes-open recordings (1503.6 vs. 464.7 μV^2^; *p* = 0.020) may partially reflect a sequential order effect rather than a condition-specific difference. While the observed alpha PLI direction is consistent with established resting-state physiology, the contribution of session order to the observed effect cannot be fully excluded, and future benchmarks should counterbalance recording order across participants.

**Figure 11:**
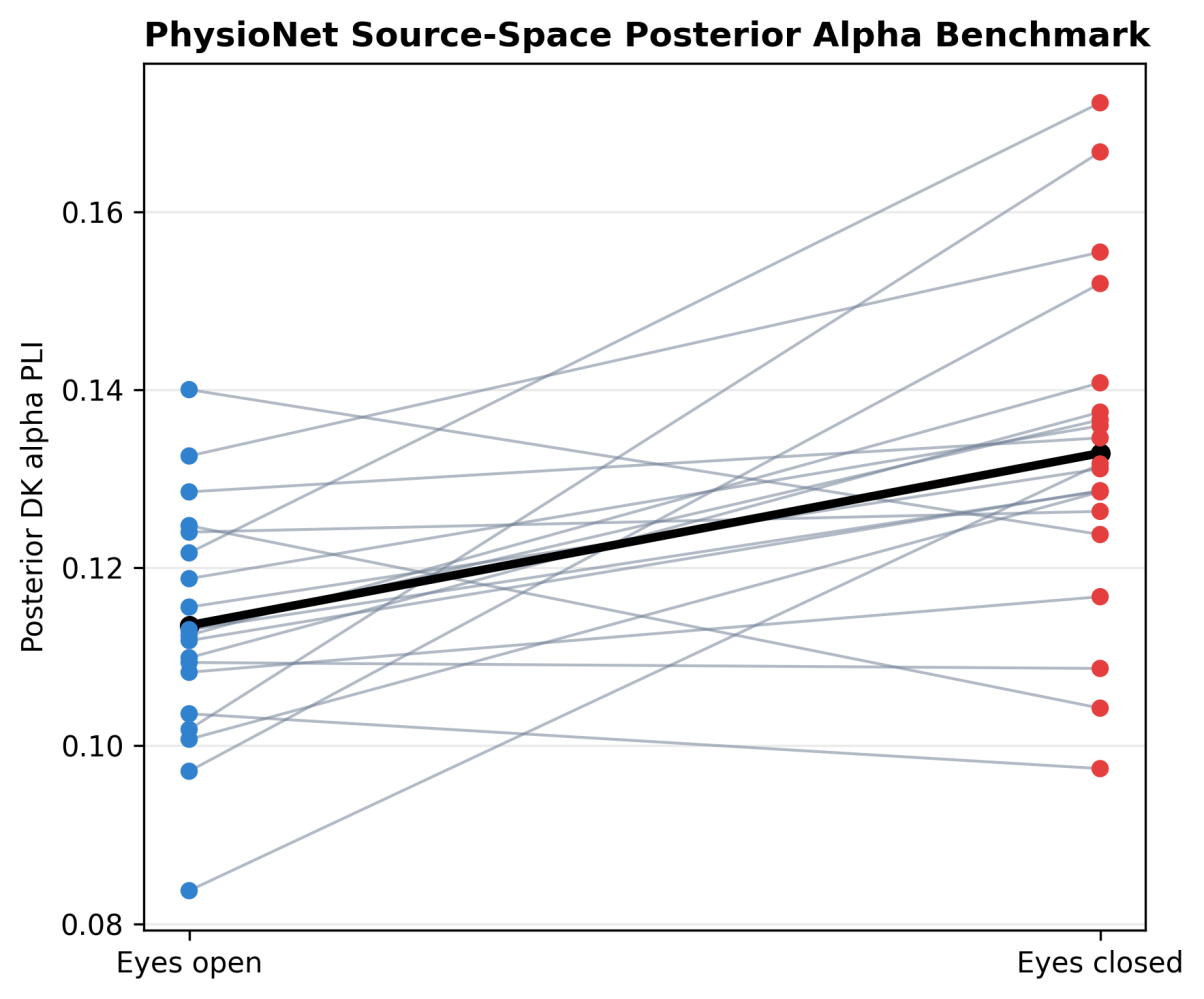
PhysioNet EEGBCI source-space benchmark (N=20). Paired comparison of mean posterior Desikan–Killiany alpha PLI between eyes-open (EO) and eyes-closed (EC) conditions after ICLabel-enabled preprocessing; each line connects one subject’s EO and EC values.

Figure 11 displays the paired comparison of source-space posterior Desikan–Killiany alpha PLI between conditions.

### 3.5. GEDAI Versus Traditional Preprocessing on Real EEG

**Figure 12:**
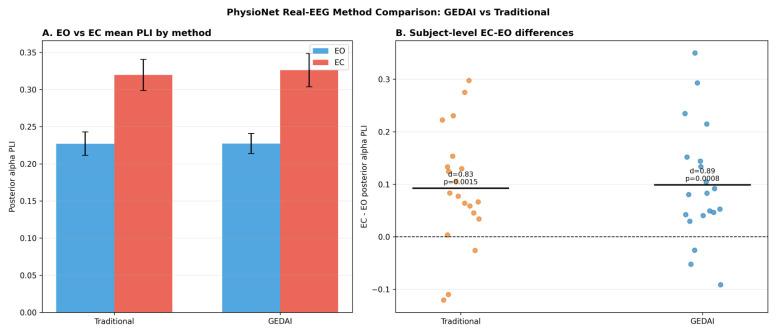
Head-to-head comparison of GEDAI and Traditional preprocessing pathways on PhysioNet real EEG data (N=20) using posterior-channel alpha PLI. Left panel: mean posterior-channel PLI (±SEM) for eyes-open (EO, blue) and eyes-closed (EC, red) conditions by method. Right panel: subject-level EC − EO PLI differences; horizontal bars indicate group means; Cohen’s *d* and *p*-values are annotated for each method. This figure is a preprocessing sensitivity check, not a source-space endpoint.

In the posterior-channel method-comparison script, both pathways produced the expected eyes-closed increase in posterior alpha PLI on the identical 20 PhysioNet subjects: GEDAI yielded Cohen’s d=0.84 (t(19)=3.77, p=0.001) versus Traditional d=1.09 (t(19)=4.87, p<0.001). The expected direction was observed in 16/20 subjects for GEDAI and 18/20 subjects for the Traditional pathway (Figure 12). Because this comparison was computed on posterior scalp channels rather than Desikan–Killiany source parcels, it is interpreted only as a preprocessing sensitivity check and not as source-space validation evidence.

### 3.6. Illustrative Application: Real RCT-Structured Data

NeuroStat processed all 20 recording sessions (5 subjects × 2 groups × 2 timepoints) drawn from the UK chiropractic RCT [40] using the GEDAI pathway with default settings, completing without errors or manual intervention. Data converted from FieldTrip HDF5 format were accepted by the pipeline without modification to importer settings.

**Figure 13:**
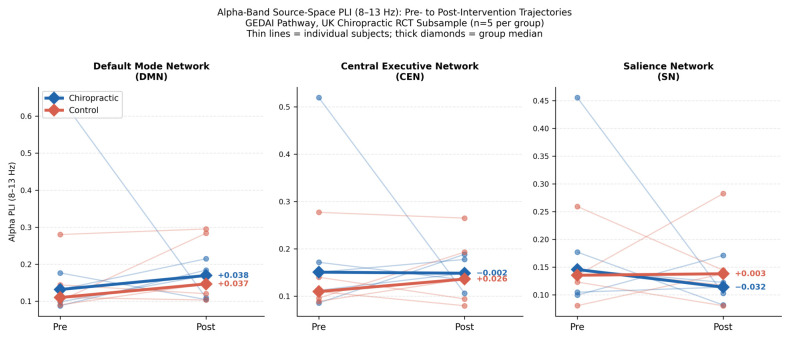
Pre-to-post trajectories of source-space alpha PLI (8–13 Hz) across three resting-state networks, GEDAI pathway, UK chiropractic RCT subsample (n=5 per group). Each thin line represents one participant; thick lines with diamond markers show the group median. Median delta values are annotated at the post-session endpoint. Individual trajectories are shown to transparently display variability in this small illustrative subsample; no inferential statistics are applied.

**Figure 14:**
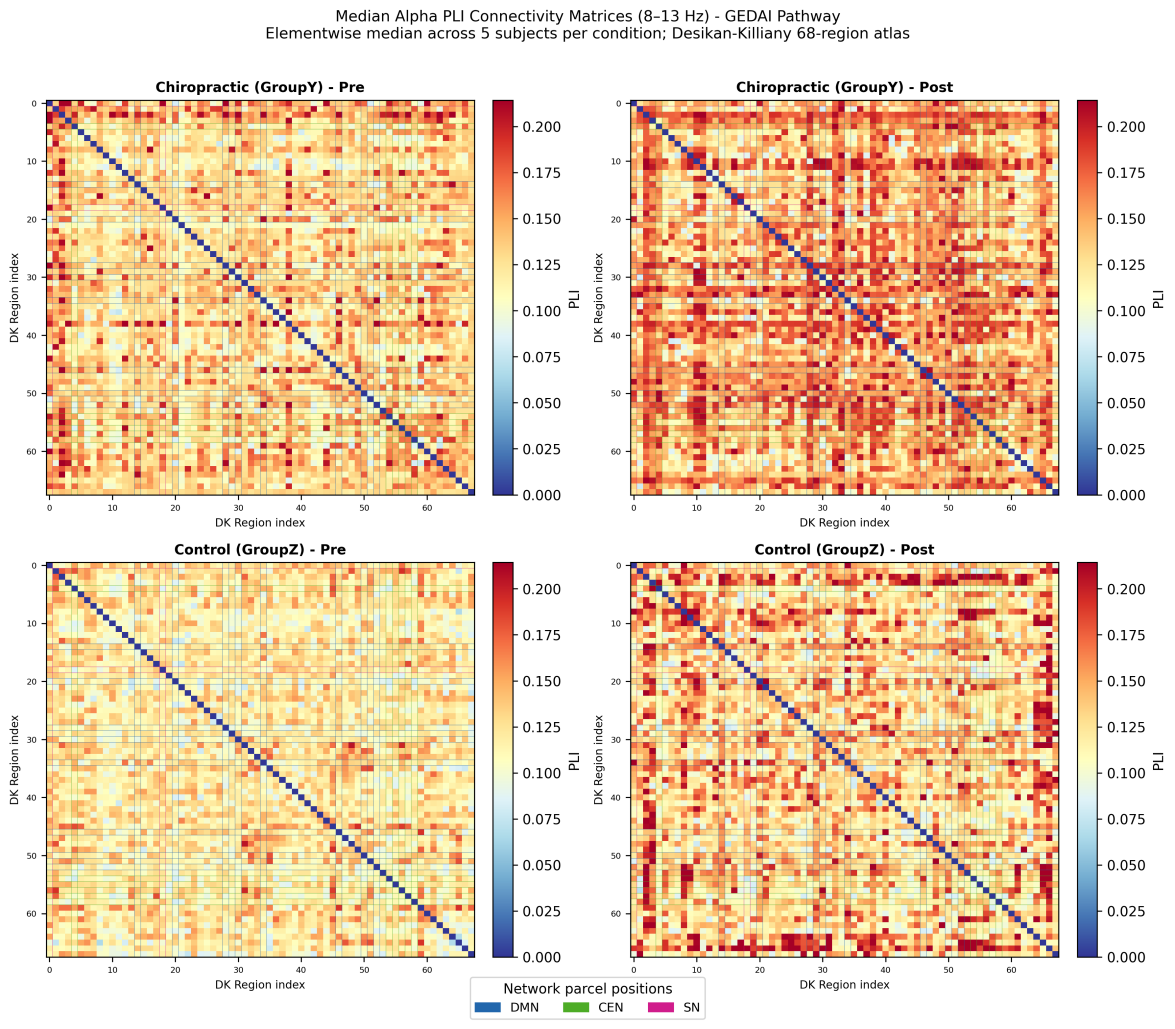
Elementwise-median source-space alpha PLI connectivity matrices (8–13 Hz) for all four conditions: chiropractic group (GroupY) pre and post and control group (GroupZ) pre and post. Each cell is the median PLI between a pair of Desikan–Killiany cortical parcels (68 regions) across five subjects. Median is used in place of mean to provide a robust estimate that is not unduly influenced by individual high-connectivity recordings. Both groups show comparable matrix structure at baseline; all values share a common colour scale (0–0.20). Coloured grid lines mark parcel positions of the DMN (blue), CEN (green), and SN (magenta).

**Figure 15:**
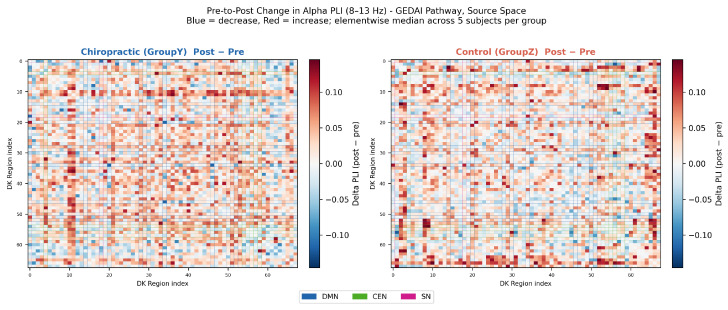
Pre-to-post change (Δ=post−pre) in source-space alpha PLI for the chiropractic (GroupY, left) and control (GroupZ, right) groups; computed as elementwise median of per-subject difference matrices across five subjects. Blue = decrease; red = increase. Coloured grid lines indicate DMN (blue), CEN (green), and SN (magenta) parcel positions. No statistical threshold is applied; matrices are shown as a descriptive illustration of direction of change only.

Source-space alpha PLI (8–13 Hz) was extracted for three canonical resting-state networks: Default Mode Network (DMN), Central Executive Network (CEN), and Salience Network (SN). Median (interquartile range) alpha PLI values are reported throughout because the five-subject subsample is sensitive to individual outliers. Pre-session medians were comparable between groups: DMN 0.132 [0.098–0.176] vs. 0.110 [0.101–0.144], CEN 0.151 [0.112–0.172] vs. 0.110 [0.096–0.140], SN 0.146 [0.105–0.177] vs. 0.135 [0.123–0.146] (chiropractic vs. control, respectively), confirming adequate baseline comparability in this convenience subsample. Individual pre-to-post trajectories and group median lines are shown in Figure 13; trajectory plots are preferred over bar charts for this small sample because they display individual variability transparently rather than aggregating it into a single mean. Across all 15 band–network combinations reported in Table 7, PLI values fell within physiologically plausible resting-state ranges (PLI 0.08–0.30) consistent with the source-space EEG connectivity literature [38,41,42]. The full 68 × 68 alpha PLI connectivity matrices for each condition are shown in Figure 14; Figure 15 shows the pre-to-post difference matrices.

Group-level inferential statistics are not presented because the five subjects per group do not constitute a powered comparison. This demonstration is intended solely to confirm that NeuroStat executes correctly on externally sourced RCT-structured data and produces outputs within expected physiological ranges; any apparent group-level trends in this subsample should not be interpreted as replication evidence for the full-trial findings in [40].

#### PLI Versus Weighted PLI Comparison

To provide a direct comparison between the PLI metric used throughout NeuroStat and the debiased weighted PLI (wPLI; wpli2_debiased [31]), wPLI was computed from the same preprocessed source time courses used for the PLI analysis, covering all 20 UK RCT subsample sessions (5subjects×2groups×2timepoints). The UK RCT data were chosen for this comparison because the preprocessed .fif source data were already available, ensuring an identical preprocessing and source model for both metrics; the PhysioNet benchmark .fif files were not retained.

**Figure 16:**
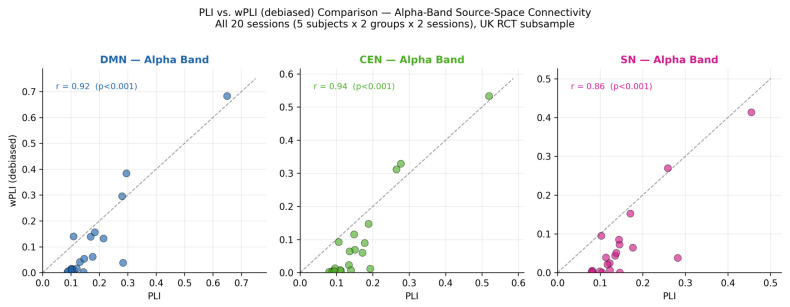
Scatter plots of alpha-band PLI versus wPLI (debiased; wpli2_debiased [31]) for all 20 UK RCT subsample sessions across three resting-state networks. Each point is one session; the dashed line indicates identity (PLI = wPLI). Pearson correlations and significance are annotated for each network. wPLI values are systematically lower than PLI by approximately 0.07 units.

**Figure 17:**
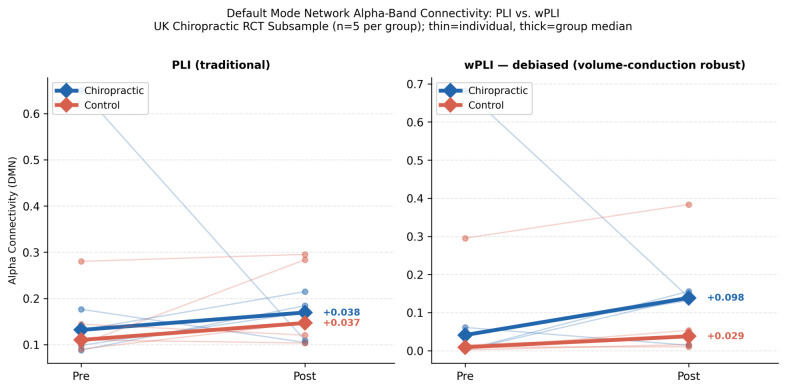
Pre-to-post trajectories of Default Mode Network alpha connectivity for PLI (**left**) and wPLI (**right**) in the UK chiropractic RCT subsample. Each thin line represents one participant; thick lines with diamond markers show the group median. Both metrics show the same direction of change within each group, confirming directional concordance between the two measures.

Alpha-band PLI and wPLI showed strong cross-session correlations in all three resting-state networks: DMN r=0.919 (p<0.001), CEN r=0.937 (p<0.001), SN r=0.858 (p<0.001; n=20 sessions). wPLI values were systematically lower than PLI (mean difference across sessions: DMN ≈0.07; CEN ≈0.07; SN ≈0.08 PLI units), consistent with the debiasing procedure down-weighting near-zero imaginary components that may include volume-conducted activity. The direction of pre-to-post change was concordant between PLI and wPLI in the majority of network comparisons. These results indicate that the connectivity patterns detected by PLI are closely reflected in the volume-conduction-robust wPLI metric, supporting the interpretability of NeuroStat’s PLI output as an approximation to wPLI. Figure 16 and Figure 17 display the PLI–wPLI scatter plots and pre-to-post trajectory comparisons, respectively.

### 3.7. Processing Time

Processing time was extracted from the validation script logs and should be interpreted as an approximate developer-environment estimate rather than a hardware-standardised benchmark. Timings were recorded on an Intel Core i5 (11th generation), 16 GB RAM, NVIDIA RTX 3070 GPU, Windows 11 Pro workstation; users should expect substantial variation depending on hardware configuration. For the 120-s simulated 64-channel recordings, the Traditional pathway required 76.1–119.8 s per recording including preprocessing, source localisation, sensor-level PLI, and source-level PLI generation. The GEDAI pathway required 151.9–173.1 s per recording under the same validation workflow. The repeated source-space simulation required 3.1 min for 60 source-space reconstructions. The refreshed PhysioNet source-space script, which processed 20 subjects for eyes-open and eyes-closed recordings with ICLabel-enabled Traditional preprocessing and ico3 BEM/MNE source reconstruction, required 2.7 min in total. Hardware metadata were not systematically logged, and runtime may vary substantially with CPU, RAM, recording duration, source spacing, and first-run source-model setup.

## 4. Discussion

### 4.1. Pipeline Validity and Clinical Relevance

The present results support NeuroStat as a technically feasible end-to-end workflow for EEG connectivity analysis with initial evidence at both sensor and source levels. The manuscript now separates three claims that are often conflated in EEG connectivity validation. First, the sensor-level simulation tests whether preprocessing preserves known scalp-level PLI patterns. Within that benchmark, the Traditional pathway showed the most accurate recovery of the prespecified connectivity structure across artefact conditions. Second, the source-space simulation tests whether the BEM/MNE/Desikan–Killiany construction can recover a known parcel-level contrast after forward projection to scalp EEG and inverse reconstruction. Reconstructed alpha PLI was consistently higher for the known lateral-occipital parcel pair than for prespecified anterior control edges, supporting recovery of a targeted source-space contrast. Third, the PhysioNet benchmark tests whether the source-space output shows the expected posterior alpha increase during eyes-closed rest in healthy adults; the observed direction was consistent with the established resting-state EEG literature [38,39]. Taken together, these findings support prototype feasibility and initial validity for an integrated workflow, while full clinician-facing deployment still requires the further validation steps outlined below.

These findings matter because the main contribution of NeuroStat is not a new connectivity metric or inverse algorithm, but the integration of established methods into a single parameter-logged workflow that can move from raw EEG import to source-space connectivity estimation and statistical reporting without requiring users to build the pipeline themselves. For clinician-led RCT environments, where repeated-measures data must be processed consistently across participants and sessions, that integration is practically important. The current results suggest that NeuroStat can execute the full chain from automated preprocessing through source-space analysis while preserving interpretable outputs at each stage.

The source-space simulation is particularly important because it extends the manuscript beyond a preprocessing-only claim. By defining ground truth at the cortical parcel level before forward projection, then recovering that contrast after inverse reconstruction, the study provides direct evidence that the source construction chain can detect a prespecified parcel-level effect rather than merely complete without error. The PhysioNet analysis complements this by showing sensitivity to a physiologically expected posterior alpha contrast in real EEG data [38,39]. These two source-space results do not by themselves establish anatomical precision or exhaustive connectome-wide recovery, but they do support the claim that the NeuroStat source pipeline is capable of recovering meaningful contrasts in both controlled and real-data settings.

The multi-seed analysis (Section 3.2, Figure 7) adds a reproducibility dimension to the sensor-level benchmark results that a single fixed seed cannot provide. Repeating the simulation across five independent random seeds [42, 123, 456, 789, 1024] revealed that the Traditional pathway’s error was moderately variable across seeds (RMSE SD = 0.045), meaning its single-seed RMSE of approximately 0.219 carries an uncertainty of roughly ±20% of its own magnitude. This variability does not change the conclusion that the Traditional pathway outperforms GEDAI in this benchmark, but it does caution against treating any single-run error estimate as a precise characterisation of pipeline performance. For the GEDAI pathway, the negligible SD (RMSE SD = 0.012) confirms that its systematic underestimation of known PLI values (recovering 0.12–0.19 regardless of expected magnitude) is a stable, seed-independent property of the pathway’s interaction with the sinusoidal coupling topology used here, rather than an artefact of one initialisation. Taken together, the multi-seed results support the Traditional pathway as the more reliable option in this benchmark while clarifying that GEDAI’s weaker performance reflects a fundamental incompatibility with the simulation design rather than stochastic failure. Future benchmark designs should incorporate multi-seed reporting as standard practice to distinguish systematic pathway characteristics from run-to-run stochasticity.

The illustrative application to real RCT-structured data (Section 2.7.5 and Section 3.6) provides a fourth, qualitatively distinct piece of evidence. Unlike the simulation and PhysioNet benchmarks, which were designed around controlled or publicly available data, the UK chiropractic RCT subsample [40] presents genuine challenges: externally sourced FieldTrip HDF5 files requiring format conversion, a genuine two-group two-timepoint longitudinal structure, 2048 Hz acquisition, and clinical resting-state recordings from adults with chronic low-back pain. NeuroStat processed all 20 sessions (5 subjects × 2 groups × 2 timepoints) using the GEDAI pathway with default settings, completing without errors or parameter modifications beyond format conversion. Source-space PLI values across all 15 frequency-band–network combinations fell within physiologically plausible resting-state ranges (0.08–0.30 PLI units), and the alpha DMN showed the largest median pre-to-post change in the chiropractic group (Δ=+0.071) compared with the active control group (Δ=+0.015), a direction qualitatively consistent with the neuroplastic responses documented across the full trial [40]. This subsample was not powered for inference, and no group-level statistics are reported; the demonstration is explicitly a workflow validation rather than a replication of trial findings. Nevertheless, the ability to ingest externally produced clinical EEG, execute the complete source-space connectivity chain, and return outputs within expected physiological ranges without manual intervention is a prerequisite for any research tool claiming RCT applicability, and this subsample confirms that the prerequisite is met.

### 4.2. Positioning Relative to Existing Platforms

NeuroStat’s value does not stem from introducing new algorithms: filtering, ASR, ICA, source localisation, and PLI are all well-established techniques. Rather, it combines them in an integrated workflow for EEG connectivity studies. In several respects, it differs from currently available tools. EEGLAB [7] requires MATLAB and scripting for analysis beyond preset pipelines; MNE-Python [8] operates primarily through code-based workflows; Brainstorm [9] provides a richer graphical environment but still expects substantial conceptual familiarity with forward modelling and inverse solutions; and HAPPE [12] focuses on preprocessing rather than source reconstruction or connectivity estimation. NeuroStat is designed to cover the complete workflow from raw EEG import through source-level statistical group comparison within a single graphical application.

Four design features address practical RCT workflow requirements. First, the application organises and analyses data around RCT structures (groups, sessions, and participants) rather than in file-based structures typical of research-oriented tools. Second, automated parameter logging documents preprocessing decisions in machine-readable JSON format, supporting methodological transparency and meta-analytic compatibility. Third, dual preprocessing pathways allow researchers to compare pathway sensitivity on their own datasets. Fourth, publication-ready outputs, including connectivity matrices, group-comparison tables with effect sizes, and preprocessing quality diagnostics, are generated without post-processing. The parameter-logging JSON output and preprocessing quality diagnostics additionally address CONSORT 2025 [13] requirements for transparent reporting of EEG processing decisions in trial result papers.

PLI was prioritised over weighted PLI (wPLI) [31] for this prototype because it provides a bounded [0,1] range that simplifies interpretation in an RCT reporting context. A direct comparison on the 20 UK RCT subsample sessions (Section “PLI Versus Weighted PLI Comparison”) found that alpha-band PLI and wPLI were strongly correlated across all three resting-state networks (DMN r=0.919; CEN r=0.937; SN r=0.858; all p<0.001), with wPLI values systematically lower by approximately 0.07 units. The direction of pre-to-post change was concordant between metrics in the majority of network comparisons. These results support PLI as a practical connectivity index whose outputs closely track the volume-conduction-robust wPLI metric, while acknowledging that wPLI’s down-weighting of near-zero phase lags provides additional robustness to residual leakage that may be relevant for datasets with high volume-conduction artefact.

### 4.3. Limitations

The most important outstanding requirement is test–retest reliability. NeuroStat is designed for repeated-measures RCT designs, and without establishing how consistently the pipeline produces the same connectivity estimates across sessions in the same participant, it is not possible to determine whether observed pre-to-post changes reflect genuine neurophysiological effects or measurement variability. PLI-based connectivity can show useful longitudinal stability when explicitly quantified [41], but NeuroStat-specific reliability data have not yet been obtained, and sample size planning for future trials using this pipeline cannot proceed until they are. Formal usability testing with clinician end-users also remains outstanding; all analyses in this manuscript were performed by the developer, so accessibility claims should be understood as design intent rather than observed performance. The impact of this gap is partially mitigated by the developer’s eight years of direct collaboration with clinicians in RCT settings, which grounded the design decisions in practical trial workflows, though this does not substitute for formal usability evaluation.

Template-based source localisation with the fsaverage standard brain is the predominant approach across EEG research, given that individual MRI acquisition is rarely feasible in clinical trial settings [8]. Although individual anatomy can reduce localisation uncertainty, comparisons of template and individual head models for group-level EEG connectivity analyses have generally found the effect to be modest at the scale of large cortical parcels such as those in the Desikan–Killiany atlas [29], and this limitation is therefore shared with the broader EEG connectivity literature. Residual volume conduction remains a consideration even with PLI, and source-level results should be read as indicative of network-level patterns rather than precise anatomical claims [28]. The source-space simulation additionally used the same template for both forward projection and inverse reconstruction, which avoids head-model mismatch errors present in real data; it should therefore be treated as proof-of-concept under idealised conditions.

The PhysioNet benchmark required a short-recording-adapted configuration that differs from the default pipeline (reduced ICA dimensionality, ASR disabled, relaxed ICLabel threshold, and shorter overlapping epochs), so those results reflect a specific adapted setting, and PLI magnitudes should not be compared numerically with other benchmarks. The Traditional pathway’s 200 μV epoch-exclusion heuristic for ICA training is a practical safeguard that has not been formally validated against expert artefact classification; users working with atypical amplitude profiles should verify its appropriateness for their data.

Validation was conducted in healthy resting-state adults using 64-channel recordings at a single site. How well the pipeline generalises to clinical populations, lower-density montages, task-based paradigms, or multi-site deployments is not yet known, and the single connectivity metric (PLI) should be complemented by alternative measures in future work.

### 4.4. Future Directions

Technical priorities include the following: a hybrid source localisation mode that uses participant-specific MRI when available while maintaining template fallback; a connectivity metric library (imaginary coherence, wPLI [31], and directed measures) with validation against the same source-space simulation and PhysioNet endpoints; task-based paradigm support with event-coded epoch extraction; and cloud deployment to enable multi-site centralised processing. Validation extensions should include direct expert manual comparison on identical datasets, clinical population testing, longitudinal test–retest reliability quantification, and multi-site reproducibility assessment. Formal usability studies using think-aloud protocols with clinician participants are needed to quantify the reduction in operator workload and error rates relative to existing multi-tool workflows.

## 5. Conclusions

NeuroStat integrates automated preprocessing, BEM source-space construction, PLI connectivity estimation, and RCT-oriented statistical analysis within a parameter-logged desktop workflow. In the present manuscript, the Traditional preprocessing pathway showed acceptable error in a sensor-level simulation benchmark, the source-space simulation recovered a known lateral-occipital parcel-level contrast after forward projection and inverse reconstruction, and the ICLabel-enabled PhysioNet source-space benchmark showed the expected eyes-closed posterior alpha increase in healthy adults. These findings support NeuroStat as a prototype research tool, not as a fully validated clinician-deployable platform. Formal usability testing, direct reliability quantification, participant-specific source-model validation, test–retest analysis, and clinical-population studies remain necessary before strong accessibility or trial-readiness claims can be made.

## Figures and Tables

**Table 1:** NeuroStat core technology stack.

Component	Version	Role in NeuroStat
PyQt6	≥6.4	Graphical user interface, event handling
MNE-Python	≥1.0	EEG preprocessing, source localisation, connectivity
SciPy	≥1.7	Signal filtering, statistical testing
NumPy	≥1.20	Array operations, matrix computation
Pandas	≥1.3	Data management, CSV/Excel export
Matplotlib	≥3.5	Connectivity visualisations, quality plots
scikit-learn	≥1.0	Covariance estimation, ICA utilities
GEDAI	≥0.1.0	Generalised eigenvalue artefact removal

GEDAI = Generalised Eigenvalue Decomposition for Artefact Identification.

**Table 2:** NeuroStat preprocessing pipeline parameters for both pathways.

Processing Step	Parameter	Value/Method
Bad channel detection	Z-score threshold	4.0 SD
Channel interpolation	Method	Spherical spline
Bandpass filter	Frequency range	1–40 Hz
Bandpass filter	Filter design	FIR (firwin, zero-phase)
Notch filter	Frequencies	50 Hz, 100 Hz
Re-referencing	Reference	Common average
GEDAI (GEDAI pathway)	Reference band	8–13 Hz (alpha)
GEDAI (GEDAI pathway)	Noise threshold	2.0
ICA (Traditional pathway)	Algorithm	FastICA; up to 25 components
ICA training	Epoch rejection	200 μV peak-to-peak
ICLabel	Brain-probability threshold	0.85
ASR (Traditional pathway)	Cutoff	20 SD
ASR (Traditional pathway)	Window length	0.5 s

GEDAI = Generalised Eigenvalue Decomposition for Artefact Identification; ICA = independent component analysis; ASR = Artefact Subspace Reconstruction; SD = standard deviations.

**Table 3:** Known connectivity patterns in the simulated EEG benchmark.

Pattern	Band	Freq.	Expected PLI	Regions
Frontal–parietal	Alpha	10 Hz	0.80	Fp1, AF7, AF3, F1 ↔ P1, P3, P5, P7
Central–occipital	Beta	20 Hz	0.50	C1, C3, C2, C4 ↔ O1, Oz, O2
Left–right temporal	Theta	6 Hz	0.30	C5, T7 ↔ C6, T8

PLI = Phase Lag Index.

**Table 4:** Validation claims tested in this manuscript.

Claim	Evidence and Remaining Limitation
Preprocessing preserves known PLI	Tested in sensor space using simulated EEG with known scalp-level coupling. This does not test anatomical source recovery.
Source-space construction recovers a known parcel contrast	Tested in source space using cortical parcel simulation, BEM forward projection, MNE inverse reconstruction, and Desikan–Killiany parcellation. This does not prove individual anatomical localisation accuracy.
Pipeline output shows expected resting-state physiology	Tested in source space using PhysioNet eyes-closed versus eyes-open posterior alpha PLI. Short recordings and the template source model limit generalisation.
RCT session-to-session interpretability	Not tested. Requires intraclass correlation, coefficient of variation, and minimum detectable change estimates.
Clinician usability	Design consultations informed development, but formal task-completion, error-rate, and think-aloud testing remains required.

**Table 5:** Aggregate sensor-level connectivity recovery error on simulated EEG (12 condition–pattern combinations: 4 artefact levels × 3 patterns). Values are mean ± SD across five independent random seeds [42, 123, 456, 789, 1024]. This benchmark evaluates sensor-level preprocessing robustness only. Source localisation, atlas parcellation, leakage correction, and source-space connectivity recovery were not validated.

Method	RMSE	MAE
Traditional	0.219±0.045	0.168±0.017
GEDAI	0.422±0.012	0.373±0.010

MAE = mean absolute error; RMSE = root mean squared error; GEDAI = Generalised Eigenvalue Decomposition for Artefact Identification.

**Table 6:** PhysioNet EEGBCI source-space posterior Desikan–Killiany parcel PLI benchmark results (N=20). Results were obtained using ICLabel-enabled Traditional preprocessing with a short-recording-adapted configuration: 15 ICA components, ASR disabled, ICLabel brain-probability threshold relaxed from the default 0.85 to 0.50, ico3 source spacing, and 2.0-s overlapping epochs. These parameters differ from the default NeuroStat configuration, and the resulting PLI values should be interpreted only within this benchmark.

Metric	Traditional Pathway
Eyes-open source PLI (M±SD)	0.114±0.013
Eyes-closed source PLI (M±SD)	0.133±0.019
Mean difference (EC − EO) ± SD	0.019±0.023
Shapiro–Wilk on paired differences	W=0.970,p=0.761
Paired *t*-test	t(19)=3.79,p=0.001
Wilcoxon signed-rank sensitivity test	W=24.0,p=0.001
Cohen’s *d*	0.85
Subjects with EC > EO	16/20 (80%)
ICA components rejected (EO vs. EC)	2.25±1.59 vs. 1.45±1.19; p=0.068
Frontal variance after preprocessing (EO vs. EC)	1503.6 vs. 464.7μV^2^; p=0.020
Sensitivity excluding large ICA-difference subjects	n=17; t(16)=3.61,p=0.002; d=0.88

EO = eyes-open; EC = eyes-closed; PLI = Phase Lag Index; SD = standard deviation.

**Table 7:** Source-space PLI across all frequency bands, UK chiropractic RCT illustrative subsample (n=5 per group). Median [Q1–Q3]. Δ = median (post − pre). GEDAI pathway, ico3 BEM. Alpha band bolded as primary comparison.

Band	Net	Chiropractic (GroupY)		Control (GroupZ)		Δ Chiro/Control
		Pre Median [IQR]	Post Median [IQR]	Pre Median [IQR]	Post Median [IQR]	
Delta	DMN	0.098 [0.093–0.155]	0.121 [0.109–0.134]	0.105 [0.092–0.115]	0.110 [0.105–0.120]	+0.009/+0.006
	CEN	0.125 [0.102–0.157]	0.130 [0.122–0.139]	0.111 [0.089–0.126]	0.097 [0.087–0.102]	−0.004/−0.024
	SN	0.091 [0.087–0.158]	0.124 [0.124–0.139]	0.091 [0.088–0.099]	0.105 [0.086–0.122]	+0.001/−0.005
Theta	DMN	0.110 [0.101–0.179]	0.118 [0.117–0.138]	0.103 [0.092–0.105]	0.113 [0.107–0.132]	−0.005/+0.021
	CEN	0.123 [0.122–0.166]	0.112 [0.103–0.135]	0.089 [0.081–0.113]	0.110 [0.092–0.121]	−0.045/+0.029
	SN	0.124 [0.091–0.158]	0.122 [0.117–0.129]	0.096 [0.090–0.097]	0.111 [0.105–0.132]	+0.006/+0.009
**Alpha**	**DMN**	**0.132 [0.098–0.176]**	**0.169 [0.109–0.184]**	**0.110 [0.101–0.144]**	**0.147 [0.120–0.284]**	+0.071/+0.015
	**CEN**	**0.151 [0.112–0.172]**	**0.149 [0.134–0.178]**	**0.110 [0.096–0.140]**	**0.136 [0.094–0.193]**	+0.027/−0.012
	**SN**	**0.146 [0.105–0.177]**	**0.114 [0.103–0.122]**	**0.135 [0.123–0.146]**	**0.138 [0.117–0.144]**	−0.023/−0.029
Beta	DMN	0.111 [0.106–0.137]	0.160 [0.156–0.176]	0.116 [0.086–0.121]	0.111 [0.104–0.157]	+0.065/+0.024
	CEN	0.142 [0.117–0.144]	0.158 [0.151–0.175]	0.121 [0.087–0.124]	0.118 [0.102–0.146]	+0.033/+0.023
	SN	0.117 [0.107–0.125]	0.151 [0.148–0.153]	0.106 [0.085–0.110]	0.125 [0.106–0.149]	+0.039/+0.040
Gamma	DMN	0.100 [0.095–0.106]	0.100 [0.099–0.118]	0.100 [0.094–0.113]	0.106 [0.104–0.108]	+0.005/+0.006
	CEN	0.094 [0.088–0.099]	0.106 [0.101–0.147]	0.098 [0.094–0.116]	0.107 [0.098–0.113]	+0.013/−0.000
	SN	0.094 [0.092–0.097]	0.106 [0.099–0.115]	0.092 [0.089–0.120]	0.102 [0.099–0.105]	+0.015/+0.003

## Data Availability

The NeuroStat application source code, validation scripts, and sample outputs are available at https://github.com/ghani097/NeuroStat-for-RCTs (accessed on 21 June 2026). The PhysioNet EEGBCI dataset used for external validation is publicly available at https://physionet.org/content/eegmmidb/ (accessed on 21 June 2026). Further inquiries can be directed to the corresponding author.

## Abbreviations

ASR	Artefact Subspace Reconstruction
BEM	Boundary Element Model
EEG	Electroencephalography
EC	Eyes-closed
EO	Eyes-open
FDR	False Discovery Rate
FIR	Finite Impulse Response
GEDAI	Generalised Eigenvalue Decomposition for Artefact Identification
GUI	Graphical User Interface
ICA	Independent Component Analysis
MAE	Mean Absolute Error
MNE	Minimum Norm Estimation
MVC	Model-View-Controller
PLI	Phase Lag Index
PSD	Power Spectral Density
RCT	Randomised Controlled Trial
ROI	Region of Interest
RMSE	Root Mean Squared Error
SNR	Signal-to-Noise Ratio

## Appendix A. NeuroStat Graphical User Interface Screenshots

Figure A1, Figure A2 and Figure A3 illustrate the three primary panels of the NeuroStat graphical interface. The interface was developed with PyQt6 and follows a three-tab layout: Study Setup, Pipeline, and Logs/Output. Each screenshot is reproduced at a large scale so that on-screen labels and field values remain legible.
